# Osimertinib Resistance in EGFR‐Mutated Non–Small Cell Lung Cancer: Mechanisms and Therapeutic Strategies

**DOI:** 10.1002/mco2.70823

**Published:** 2026-07-02

**Authors:** Junfeng Guo, Qiuyuan Wen, Songqing Fan

**Affiliations:** ^1^ Department of Pathology The Second Xiangya Hospital Central South University Changsha China; ^2^ Hunan Clinical Medical Research Center for Cancer Pathogenic Genes Testing and Diagnosis Changsha China

**Keywords:** epidermal growth factor receptor mutation (EGFR), non–small cell lung cancer, Osimertinib resistance, SWI/SNF related, matrix associated, actin dependent regulator of chromatin, subfamily A, member 4 (SMARCA4), therapeutic strategy

## Abstract

Non–small cell lung cancer (NSCLC) is a leading cause of cancer‐related deaths worldwide, with epidermal growth factor receptor (*EGFR*) mutations being one of the most common actionable genetic alterations. Osimertinib, a third‐generation EGFR tyrosine kinase inhibitor (EGFR‐TKI), has significantly improved the prognosis of patients with EGFR‐mutated NSCLC, but acquired resistance remains a major clinical obstacle limiting its long‐term efficacy. This review summarizes key mechanisms of osimertinib resistance, including both EGFR‐dependent and EGFR‐independent resistance pathways. A major focus is placed on the chromatin remodeler Switch/Sucrose nonfermentable (SWI/SNF) related, matrix‐associated, actin‐dependent regulator of chromatin, subfamily A, member 4 (SMARCA4), which mediates resistance through its interplay with EGFR signaling. Epigenetically, SMARCA4 loss modulates the tumor microenvironment and therapeutic response by triggering a stress‐induced yet functionally impaired nuclear factor erythroid 2‐related factor 2 (NRF2) antioxidant pathway and activating phosphatidylinositol 3‐kinase/protein kinase B (PI3K/AKT) signaling. This cascade not only promotes tumor proliferation and survival but also directly confers resistance to osimertinib. Beyond SMARCA4, this review also discusses bypass pathway activation and further summarizes recent advances in novel targeted agents and combination therapies against osimertinib resistance. Overall, it provides an integrated overview of resistance mechanisms and supports the development of improved precision therapies for EGFR‐mutant NSCLC.

## Graphical abstract

This review illustrates the key mechanisms of osimertinib resistance in EGFR‑mutant NSCLC, including EGFR‑dependent mutations, bypass pathway activation, and SMARCA4‑mediated epigenetic regulation. We also summarize emerging targeted therapies and future perspectives to overcome EGFR‑TKI resistance.

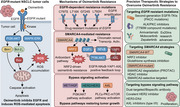

## 1 Introduction

Accounting for 11.4% of global cancer incidence and 18.0% of cancer mortality, non–small cell lung cancer (NSCLC) is a major target for precision oncology optimization [1]. Epidermal growth factor receptor (*EGFR*) mutations are detected in approximately 50.3% of East Asian patients and 34.2% of South Asian patients, compared with 26.8% of admixed American, 14% of African, and 13% of European patients, making EGFR tyrosine kinase inhibitors (EGFR‐TKIs) the cornerstone of targeted therapy for this subgroup [2, 3]. Osimertinib, a third‐generation EGFR‐TKI, selectively targets *EGFR*‐sensitive mutations (e.g., exon 19 deletions, L858R point mutations) and the T790M resistance mutation, substantially improving median progression‐free survival (PFS) and overall survival (OS) in patients with EGFR‐sensitizing mutations. However, several real‐world studies demonstrate that resistance to osimertinib emerges at approximately 17–18 months with first‐line treatment and at 10–14 months with second‐line treatment, severely limiting the clinical benefit of osimertinib [4, 5].

Osimertinib resistance is a complex, multifactorial process, generally classified into EGFR‐dependent resistance (e.g., secondary *EGFR* mutations) and EGFR‐independent resistance (e.g., bypass‐pathway activation) [6]. Among the various resistance mechanisms, SWI/SNF‐related, matrix‐associated, actin‐dependent regulator of chromatin, subfamily A, member 4 (SMARCA4) has recently emerged as a key regulator that interfaces with EGFR signaling to mediate osimertinib resistance. *SMARCA4* inactivating mutations (found in 15%–20% of lung adenocarcinomas and 3%–5% of lung squamous cell carcinomas) impair essential chromatin remodeling, resulting in aberrant chromatin accessibility and constitutive activation of the EGFR pathway [7, 8]. Consequently, SMARCA4‐deficient tumors show pronounced resistance to osimertinib. Functional cell line experiments demonstrated that *EGFR* wild‐type*, SMARCA4‐*mutant A549 cells were resistant to EGFR‐TKIs. Restoration of wild‐type *SMARCA4* expression successfully resensitized these cells to EGFR‐TKIs both in vitro and in vivo. Conversely, silencing of endogenous wild‐type *SMARCA4* in H358 cells conferred a trend toward resistance to EGFR‐TKIs [9]. With ongoing advancement of research, an increasing number of novel resistance mechanisms and therapeutic targets have been identified, providing new opportunities to overcome osimertinib resistance in SMARCA4‐mutant NSCLC.Table S1

This review focuses on the mechanisms of osimertinib resistance in *EGFR*‐mutated NSCLC, comprehensively elaborates on the role of SMARCA4 and other key molecules/pathways in resistance development, summarizes the latest therapeutic strategies and research advancements, and discusses future perspectives, aiming to provide a theoretical basis for precision clinical treatment of osimertinib‐resistant *EGFR*‐mutated NSCLC.

## 2 EGFR Mutations and Mechanisms of Osimertinib Action in NSCLC

EGFR mutations are critical oncogenic drivers in NSCLC, with distinct subtypes conferring differential therapeutic responses. As a third‑generation irreversible EGFR‑TKI, osimertinib is the standard of care for EGFR‑mutant NSCLC, and its antitumor mechanisms involve multiple signaling and apoptotic pathways. Therefore, elucidating the signaling cascades underlying osimertinib‑induced tumor cell death provides a mechanistic basis for its therapeutic efficacy and the development of resistance.

### 2.1 EGFR Mutations in NSCLC

The epidermal growth factor receptor (EGFR/HER1) belongs to the receptor tyrosine kinase (RTK) family, which also includes HER2 (ErbB2), HER3 (ErbB3), and HER4 (ErbB4), and plays a critical role, together with HER2, in NSCLC progression [10, 11, 12]. EGFR activation is initiated by ligand binding (e.g., epidermal growth factor [EGF]) to the extracellular domain, which induces receptor dimerization (homodimers or heterodimers with other ErbB receptors) [13]. This leads to conformational changes that promote asymmetric dimer formation, thereby activating the intracellular kinase domain and triggering autophosphorylation of C‐terminal tyrosine residues [14]. These phosphorylation events activate key downstream pathways, including phosphatidylinositol 3‐kinase/protein kinase B/mammalian target of rapamycin (PI3K/AKT/mTOR) pathway, the mitogen‐activated protein kinase/extracellular signal‐regulated kinase (MAPK/ERK) pathway, and the Janus kinase/signal transducer and activator of transcription (JAK/STAT) pathway, thereby governing cell proliferation, differentiation, migration, and resistance to apoptosis [15]. EGFR activity is regulated by the dynamic transition between inactive and active kinase conformations [16]. Domain‐specific *EGFR* mutations (extracellular, transmembrane, or intracellular) disrupt these regulatory mechanisms, resulting in constitutive tyrosine phosphorylation and pathological signaling dysregulation.

Most EGFR activating mutations are located within the tyrosine kinase domain (exons 18–21) [17, 18]. The European Society for Medical Oncology (ESMO) guidelines classify exon 19 in‐frame deletions (ex19del, 45%) and exon 21 L858R point mutation (ex21 L858R, 40%) represent the most common mutations represent the most common mutations in *EGFR*‐mutant NSCLC, whereas exon 20 in‐frame insertions (ex20ins, 4%–10%) are less common. Less common mutations include exon 19 insertions (ex19ins), G719X in exon 18, L861Q in exon 21, S768I in exon 20, and T790M in exon 20 [19]. These mutations induce conformational changes that trigger ligand‐independent autophosphorylation of tyrosine residues, leading to constitutive activation of EGFR signaling and sustained tumor growth.

In addition to activating mutations, secondary *EGFR* mutations (e.g., T790M, C797S, L792F) represent key drivers of acquired resistance to EGFR‐TKI. Among these, T790M is the predominant resistance mutation against first‐ and second‐generation agents, detected in approximately 50% of cases [6]. Although osimertinib was designed to overcome T790M‑mediated resistance, prolonged treatment frequently leads to the emergence of additional secondary mutations (e.g., C797S) [20]. Notably, L858R mutations often coexist with noncommon EGFR mutations, which may partly explain the attenuated efficacy of osimertinib in patients with L858R‐mutant tumors compared with those harboring ex19del. Conversely, the co‐occurrence of uncommon mutations is far less frequent in patients with ex 19del. However, these patients exhibit a higher propensity to develop C797S mutations as an acquired resistance mechanism [21].

### 2.2 Mechanisms of Osimertinib‐Induced Tumor Cell Death

Under physiological conditions, EGFR stimulates nuclear factor erythroid 2‐related factor 2 (NRF2) signaling to mitigate oxidative stress by clearing intracellular reactive oxygen species (ROS) and promotes cell survival through anti‐apoptotic signals mediated by the ERK/Bim (Bcl‐2‐interacting mediator of cell death) axis [22, 23]. This occurs through two main mechanisms: (i) PI3K/AKT pathway activation, wherein AKT‐mediated phosphorylation facilitates NRF2 nuclear translocation while concurrently inhibiting glycogen synthase kinase 3β (GSK‐3β) and Kelch‐like ECH‐associated protein 1 (KEAP1), thereby reducing NRF2 ubiquitination and degradation; (ii) MAPK/ERK pathway activation: EGFR phosphorylation recruits Grb2, leading to ERK‐mediated phosphorylation of activator protein 1 (AP‐1) (e.g., c‐Fos/c‐Jun) and E26 transformation‐specific (ETS) transcription factors (e.g., ELK‐1), which in turn enhance NRF2 transcription [24, 25, 26]. In parallel, under basal EGFR signaling, constitutively active ERK also phosphorylates Bim, targeting it for ubiquitin‐dependent proteasomal degradation and thus suppressing apoptotic signaling to maintain cancer cell survival [27]. Under oxidative stress, nuclear NRF2 binds to antioxidant response elements (AREs), upregulating detoxification enzymes to restore redox balance. Low levels of ROS further augment NRF2 activity by promoting KEAP1 oxidation or stimulating the PI3K/AKT pathway, thereby reinforcing antioxidant defenses [28].

Osimertinib, an irreversible third‐generation EGFR‐TKI, exerts its therapeutic effect in EGFR‐mutant NSCLC primarily through the inhibition of EGFR tyrosine kinase activity. This inhibition prevents autophosphorylation and suppresses the downstream PI3K/AKT and MAPK/ERK signaling cascades [29]. Reduced ERK activity diminishes phosphorylation at the Ser69 residue of pro‐apoptotic BH3‐only protein Bim, thereby preventing its ubiquitin‐mediated proteasomal degradation and leading to Bim stabilization and upregulation [30]. This Bim‐centered regulation, in conjunction with osimertinib‑induced alterations in classic apoptotic regulators—downregulation of anti‐apoptotic Bcl‐2 and upregulation of pro‐apoptotic Bcl‐2‐associated X protein (Bax)—promotes Bim‐dependent sequestration of Bcl‐2, thereby liberating Bax to translocate to mitochondria and synergistically inducing mitochondrial outer membrane permeabilization (MOMP) [31, 32]. MOMP results in the collapse of the mitochondrial membrane potential, the release of cytochrome c, and the activation of caspase cascades, ultimately driving cancer cell apoptosis. Moreover, dying tumor cells produce ROS, which activates the c‐Jun N‐terminal kinase (JNK) pathway. Activated JNK not only accelerates Bax translocation to mitochondria but also further suppresses ERK activity, thereby strengthening Bim stabilization and augmenting apoptotic signaling through a positive feedback loop [33]. Consequently, this mechanism also disrupts the tumor microenvironment [34].

**FIGURE 1:**
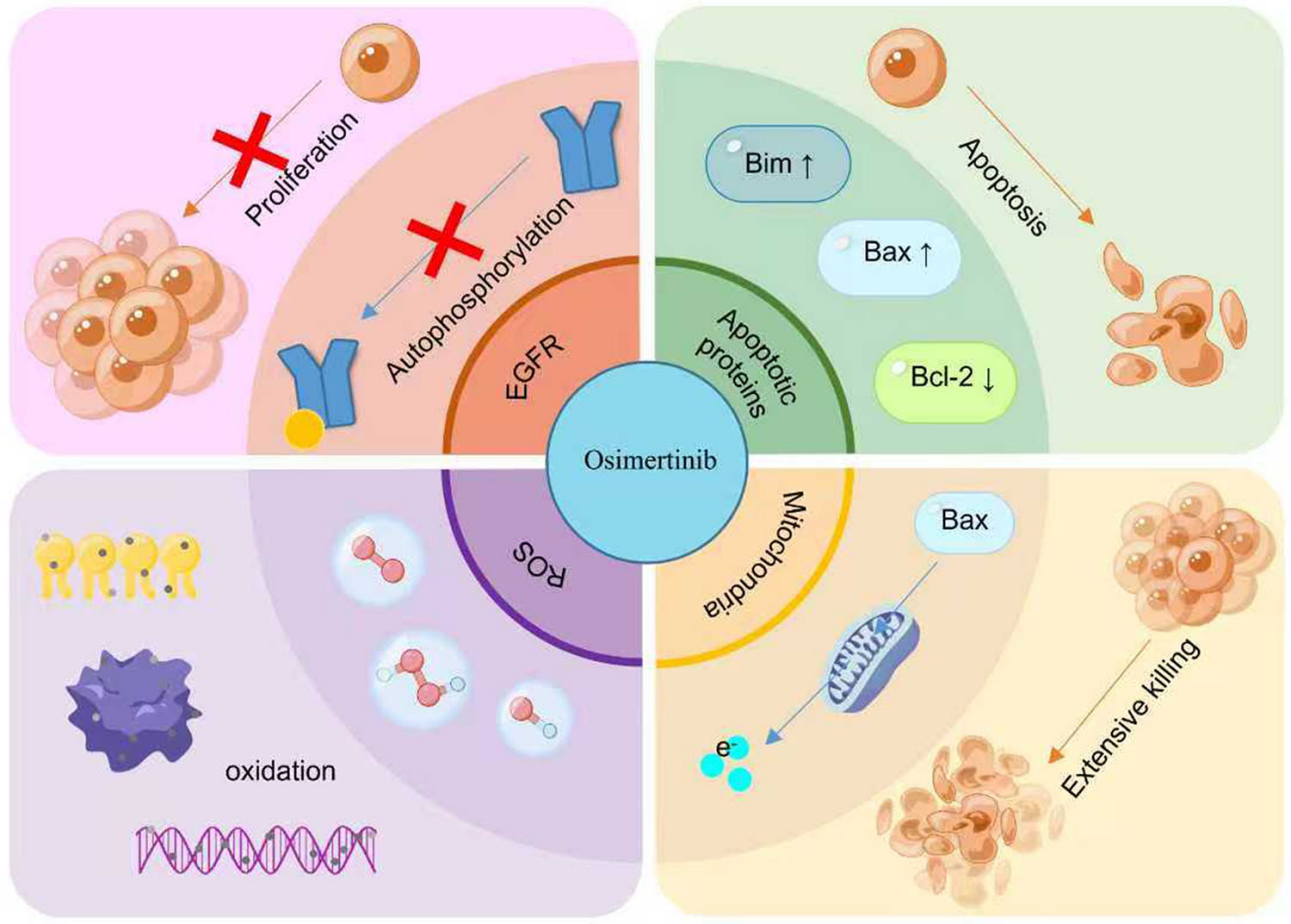
Osimertinib mediates cytotoxicity through four key actions. Inhibition of EGFR autophosphorylation halts proliferation; imbalanced expression of pro‐/anti‐apoptotic proteins facilitate apoptosis; mitochondrial dysfunction impairs energy production, enhancing cell death; and resultant ROS accumulation damages lipids, proteins, and nucleic acids via oxidation.

EGFR inhibition triggers intracellular accumulation of ROS and oxidative injury, ultimately resulting in tumor cell death (**Figure** **1**). Attenuation of PI3K/AKT and ERK/Bim signaling impairs NRF2 activity, thereby suppressing antioxidant gene expression and ROS detoxification. Mitochondrial dysfunction, arising from an imbalance between pro‐apoptotic/anti‐apoptotic proteins, disrupts the electron transport chain, leading to elevated production of superoxide anions and other ROS [35, 36]. This dysfunction further impairs energy metabolism, critically affecting tumor cells that are highly dependent on efficient energy supply. Elevated ROS oxidize lipids, proteins, and nucleic acids, resulting in membrane lipid peroxidation, protein dysfunction, and DNA lesions [37]. The subsequent activation of p53 induces cell cycle arrest and apoptosis, mediating the cytotoxic effects of osimertinib.

## 3 EGFR‐Dependent Resistance Mechanisms in EGFR‐Mutated NSCLC

Osimertinib resistance is a multifactorial process driven by diverse molecular mechanisms, which can be broadly categorized into two main types: EGFR‐dependent resistance and EGFR‐independent resistance. EGFR‑dependent resistance primarily stems from acquired alterations within the *EGFR* gene, notably secondary mutations and gene amplification.

The C797S mutation within the EGFR tyrosine kinase domain is the most frequent secondary alteration driving osimertinib resistance. By abolishing the covalent interaction between osimertinib and EGFR, this mutation constitutes the principal resistance mechanism against third‐generation EGFR‐TKIs. Comprehensive genomic profiling of osimertinib‐resistant NSCLC has identified C797S as the predominant on‐target EGFR resistance mutation, present in 66% of cases, followed by less common secondary mutations, including L718X (14%), G724S (11%), and L792F (2.2%) [38, 39]. Similar to C797S, these rare variants also reduce osimertinib sensitivity through structurally distortion of the EGFR kinase domain. Notably, these resistance‐associated mutations frequently occur concurrently with one another or with the T790M alteration, giving rise to complex genotypes that demand individualized therapeutic strategies.

*EGFR* amplification represents another key EGFR‐dependent resistance mechanism, whereby increased gene copy number drives EGFR protein overexpression, counteracting the osimertinib‑mediated inhibition of EGFR tyrosine kinase activity. Evidence indicates that the frequency of EGFR amplification in osimertinib‐resistant patients differs according to treatment context and detection methodology, typically falling between ranging and 5% to 10% in the overall osimertinib‐resistant population. Notably, recent prospective clinical cohort studies have yielded more refined estimates. A large‐scale multicenter prospective study enrolled 149 patients with advanced EGFR‐mutant NSCLC who progressed on first‐line osimertinib identified *EGFR* amplification in 32.9% (49/149) of tissue specimens [40].

## 4 EGFR‐Independent Resistance Mechanisms Driven by SMARCA4 in EGFR‐Mutated NSCLC

EGFR‐independent resistance constitutes an alternative mode of osimertinib resistance, arising from activation of bypass signaling pathways, epigenetic dysregulation, alterations in the tumor microenvironment, enrichment of cancer stem cells (CSCs), and histological transformation. This section focuses on SMARCA4‐mediated epigenetic dysregulation.

### 4.1 Functions of Chromatin Remodeling Complexes Localization

**FIGURE 2:**
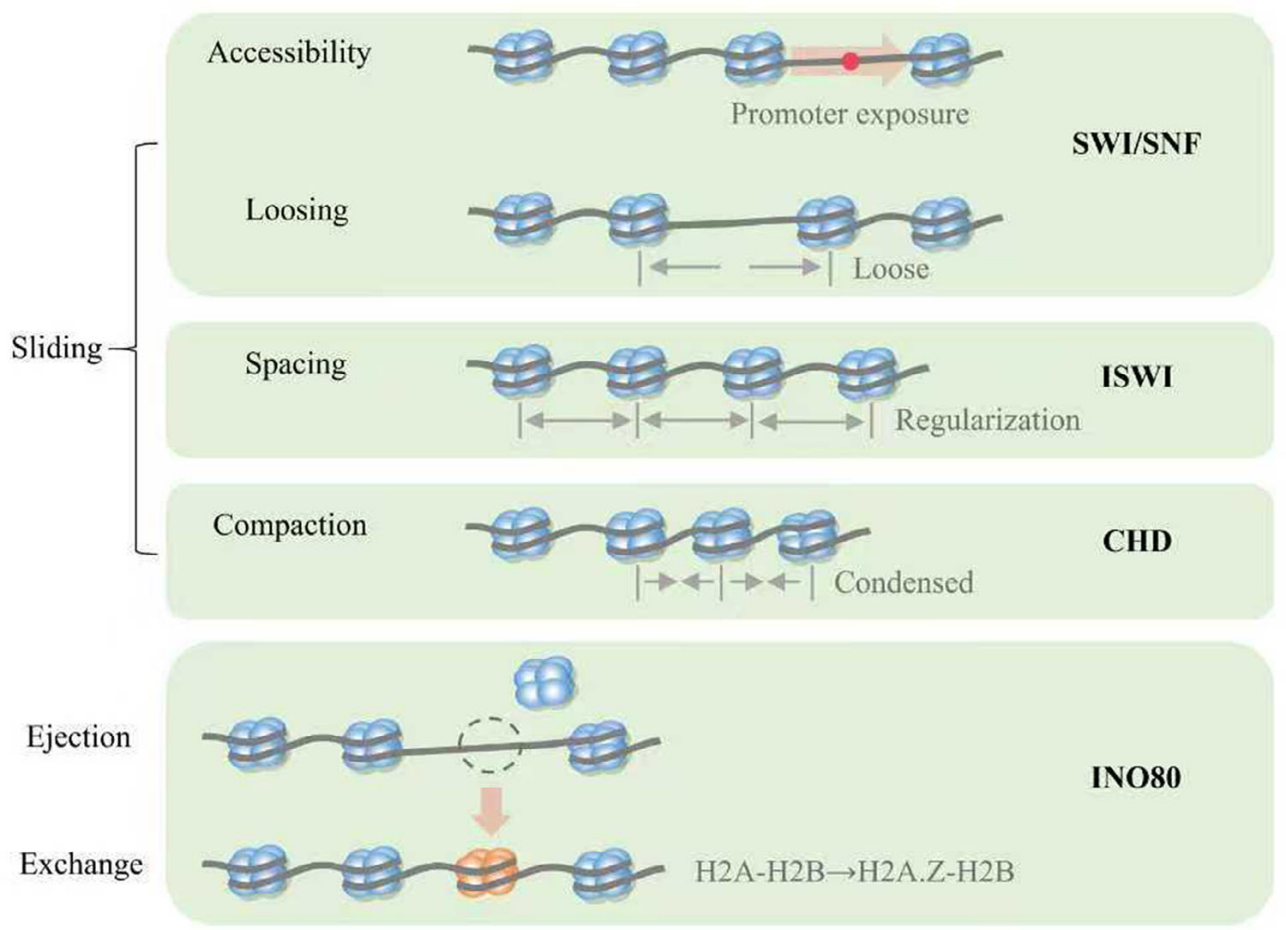
CRCs govern gene expression by altering chromatin accessibility via nucleosome sliding, ejection, and histone exchange. Key ATP‐dependent CRC families achieve this through divergent strategies: SWI/SNF complexes employ BRG1/BRM subunits to slide nucleosomes, exposing regulatory sites for gene activation; ISWI complexes use SNF2L to standardize nucleosome spacing, compacting chromatin to inhibit transcription; CHD family proteins (CHD1‐4) recognize histone modifications to compact nucleosomes and enforce silencing; and the INO80 complex initiates DNA repair by replacing H2A‐H2B with H2A.Z variants to attract repair factors.

Chromatin remodeling complexes (CRCs) are multiprotein assemblies that dynamically regulate chromatin architecture and DNA accessibility. They are classified into four families based on their catalytic ATPase subunits: SWI/SNF, ISWI, CHD, and INO80/SWR [41, 42]. Packaging of DNA into nucleosomes establishes transcriptionally repressive heterochromatin. CRCs counteract this repression through ATP‐ dependent remodeling mechanisms (e.g., nucleosome sliding, ejection, or exchange), thereby increasing DNA accessibility for transcription factors (**Figure** **2**). The SWI/SNF complex (termed BAF in mammals) is frequently altered in human malignancies (20%–25% of tumors) and employs BRG1/BRM ATPases to modulate gene expression [43]. CHD‐family complexes, such as the NuRD complex containing CHD3/CHD4, bind methylated histone lysines and acetylate histone deacetylation to modulate chromatin states. INO80 facilitates transcription and DNA repair through nucleosome repositioning and histone variant incorporation, whereas ISWI maintains heterochromatin integrity by regulating nucleosome spacing. Dysregulation of both families has been linked to oncogenesis. This epigenetic network balances chromatin dynamics: SWI/SNF generates nucleosome‑free regions (NFRs) via nucleosome sliding, conferring structural plasticity; INO80 promotes plasticity through exchange of H2A‑H2B dimers. In contrast, CHD enforces stability by compacting nucleosomes to promote regular spacing, and ISWI silences gene promoters by establishing ordered nucleosome arrays [44, 45, 46, 47]. ATPase‐ dependent control of nucleosome organization by these complexes governs genomic accessibility and cellular differentiation [47, 48, 49, 50, 51, 52, 53, 54, 55, 56, 57] (**Table** **1**). Disruption of CRC subunits by mutation compromises complex integrity, causes genomic mislocalization, and induces transcriptional chaos that drives tumor initiation. Aberrant chromatin remodeling further reprograms the tumor microenvironment through dysregulation of stromal genes, promoting metastatic progression.

**TABLE 1:** Classification, functions, and ATPase subunits of chromatin remodeling complexes.

CRCs^a^	Activating‐type^b^		Conservative‐type^c^	
	SWI/SNF	INO80	CHD	ISWI
Representative complexes	BAF, PBAF^d^ ncBAF^e^	INO80 complex SWR1 complex	NuRD	ACF, RSF NoRC
Function	Accessibility/Losing^f^	Ejection/Exchange^g^	Compaction^h^	Spacing^i^
ATPase subunit	BRG1, BRM	INO80, P400 SRCAP	CHD1‐4	SNF2L SNF2H

^a^ CRCs: chromatin remodeling complexes.

^b^ Activating‐type: promote chromatin accessibility.

^c^ Conservative‐type: reduce chromatin accessibility.

^d^ PBAF: polybromo‐associated BAF complex

^e^ ncBAF: noncanonical BAF complex

^f^ Accessibility/Losing: nucleosome sliding leads to a looser structure and the generation of nucleosome‐free regions (NFRs).

^g^ Ejection/Exchange: exchange H2A‐H2B dimmers for H2A.Z‐H2B via ejection.

^h^ Compaction: compaction of nucleosome gaps leads to chromatin condensation.

^i^ Spacing: turn off the gene promoter region with spacing.

Serving as the catalytic ATPase of the SWI/SNF chromatin remodeling complex, SMARCA4 facilitates transcription factor binding to DNA by modulating nucleosome architecture and histone conformation, thereby promoting transcriptional activation [58, 59]. This protein plays a critical role in governing cell proliferation, differentiation, and apoptosis. Although SMARCA4 is crucial for preserving genomic stability, its inactivation contributes to oncogenesis across a wide spectrum of cancers. Aberrations in SMARCA4, including homozygous deletions, gene fusions, and truncating mutations, cause protein loss, increased tumor mutational burden (TMB), and c are associated with unfavorable prognosis [60]. Loss of SMARCA4 function hyperactivates EGFR signaling by disrupting chromatin accessibility and repressing pathway inhibitors, thus promoting tumor proliferation and survival [61]. Simultaneous dysregulation of cooperating oncogenes further augments tumor invasiveness and is predictive of adverse clinical outcomes.

### 4.2 Functions and Mutations of the Human *SMARCA4*

The human *SMARCA4* gene maps to chromosomal region 19q13.2 and spans approximately 45kb of genomic DNA [62]. It encodes the SMARCA4 protein (BRG1), the core catalytic ATPase of the SWI/SNF chromatin remodeling complex. This multi‐domain protein contains eight evolutionarily conserved functional regions: (1) an ATPase domain that drives chromatin remodeling through ATP hydrolysis; (2) a bromodomain that recognizes acetylated histone lysines facilitating chromatin restructuring and transcriptional control; (3) C2H2 zinc fingers: Enable sequence‐specific DNA recognition; (4) a Helicase/SANT (SWI2/SNF2) domain that mediates interactions between nucleosomal histones and‐DNA; (5) a HSA domain (Helicase‐SANT‐associated) domain responsible for coordinates protein–protein assembly within SWI/SNF; (6) a BRK domain (Brahma/Kismet‐like) domain that governs complex specificity and assembly; (7) a nuclear localization signal (NLS) that directs nuclear translocation; and (8) transcription factor interaction domains that recruit SMARCA4 to genomic loci and interact with transcriptional regulators to modulate gene expression [63, 64].

SMARCA4 has emerged as a central focus in both developmental biology and cancer research. Through its ATPase activity, the SWI/SNF complex hydrolyzes ATP to remodel chromatin, thereby increasing DNA accessibility for gene regulatory complexes, DNA repair machinery, and transcriptional coactivators [65]. This remodeling exposes regulatory genomic elements, promotes the recruitment of transcription factors to their cognate sites, and stimulates gene expression. Conversely, SMARCA4‐dependent chromatin reorganization can repress transcription by preventing transcription factor binding or displacing RNA polymerase. These dual regulatory functions are essential for embryogenesis, cellular differentiation, and stem cell maintenance. SMARCA4 further governs G1/S phase progression via the PI3K/AKT and MAPK signaling. Importantly, SMARCA4 deficiency compromises the integrity of all SWI/SNF complex variants, impairing chromatin binding and opening at regulatory regions [22]. Lung lineage‑specific transcription factors direct alveolar development, differentiation, and homeostasis by engaging specific chromatin regions to control genes critical for pulmonary function. Mutations in *SMARCA4* induce chromatin compaction and restrict DNA accessibility, thereby hindering transcription factor occupancy at target loci. This dysregulation promotes tumorigenesis through the disruption of normal cellular processes. Moreover, mutant *SMARCA4* fails to evict polycomb repressive complexes (PRCs), resulting in their aberrant accumulation at gene promoters. The consequent deregulation of PRC activity drives tumor cell proliferation and invasion.

SMARCA4 is the most frequently mutated SWI/SNF ATPase in human cancers, and its inactivation has been implicated in malignancies, including lung, breast, and colorectal cancer [66]. These mutations fall into two classes: Class I comprises truncating or loss‐of‐function (LoF) variants (e.g., nonsense, frameshift, splice‐site, or large deletion mutations), whereas Class II encompasses all missense variants, which are further subdivided into likely pathogenic variants and variants of unknown significance (VUS) [67]. Cryo‐EM structural studies of the human SWI/SNF complex reveal that oncogenic mutations cluster at interfaces between subunits. These interaction interfaces serve as binding sites for regulatory factors or nucleosomes, indicating that mutations impair chromatin remodeling by disrupting these contacts [68]. SMARCA4 alterations are linked to multiple cancer types characterized by high TMB and a scarcity of actionable drivers. For instance, *SMARCA4* mutation constitutes the hallmark genetic lesion in small cell carcinoma of the ovary, hypercalcemic type (SCCOHT), a rare malignancy primarily affecting young women and exhibiting small‐cell/rhabdomyosarcomatous morphology [69]. In NSCLC, *SMARCA4* mutations are frequently associated with sarcomatoid differentiation and rarely co‐occur with *EGFR* mutations, suggesting that they play distinct biological roles in molecularly defined NSCLC subgroups, thereby influencing tumor behavior and therapy response [70]. Consequently, SMARCA4 represents a candidate therapeutic target in both *SMARCA4*‐mutant NSCLC, including osimertinib‐resistant tumors, and *SMARCA4* wild‐type NSCLC harboring *EGFR* mutations. Notably, the core biological functions of SMARCA4, including chromatin remodeling and regulation of cell proliferation, can be either abolished by loss‐of‐function genetic mutations or precisely modulated by multiple intracellular signaling pathways. This dual regulation of SMARCA4, exerted at both the genetic level and through signal‐mediated posttranscriptional/protein‐level mechanisms, provides a critical foundation for further elucidating its signaling regulatory network and its intricate crosstalk with EGFR signaling in NSCLC.

### 4.3 Signaling Networks Regulating SMARCA4 Activity

**FIGURE 3:**
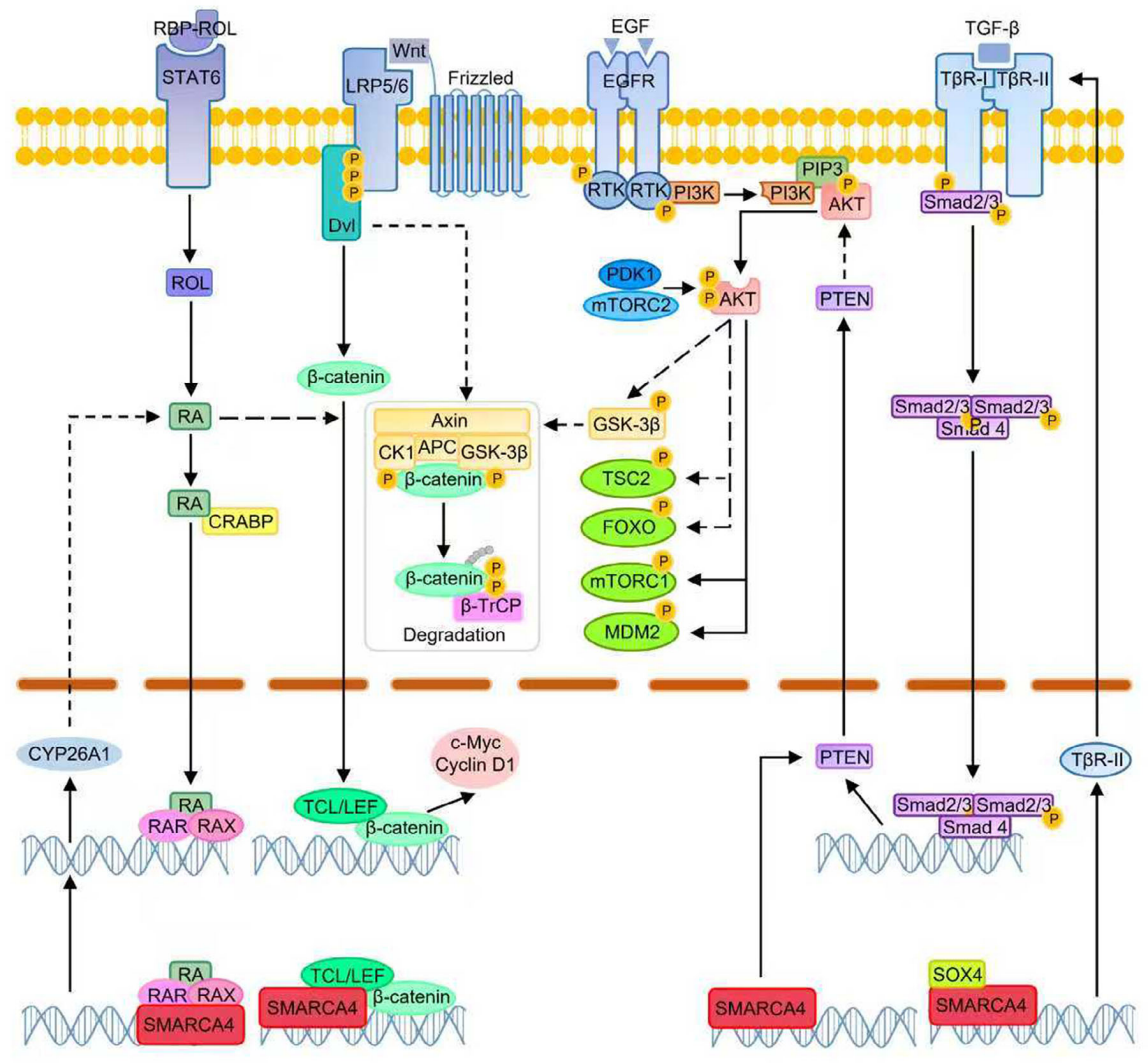
SMARCA4 and crosstalk occurs among the RA, Wnt/β‑catenin, and PI3K/AKT pathways. Activated AKT phosphorylates downstream effectors (including GSK‐3β, mTORC1, MDM2, TSC2, and FOXO). GSK‐3βinterfaces with the Wnt/β‐catenin signaling, while AKT directly engages the TNFα/NFκB pathway. SMARCA4 modulates RA signaling through RAR/RXR complex‐mediated chromatin remodeling and transcriptional activation. This pathway exhibits feedback crosstalk with the Wnt/β‐catenin signaling. SMARCA4 enhances Wnt target genes expression either by direct β‐catenin binding or by functioning as an ATP‐dependent chromatin remodeling coactivator.

**FIGURE 4:**
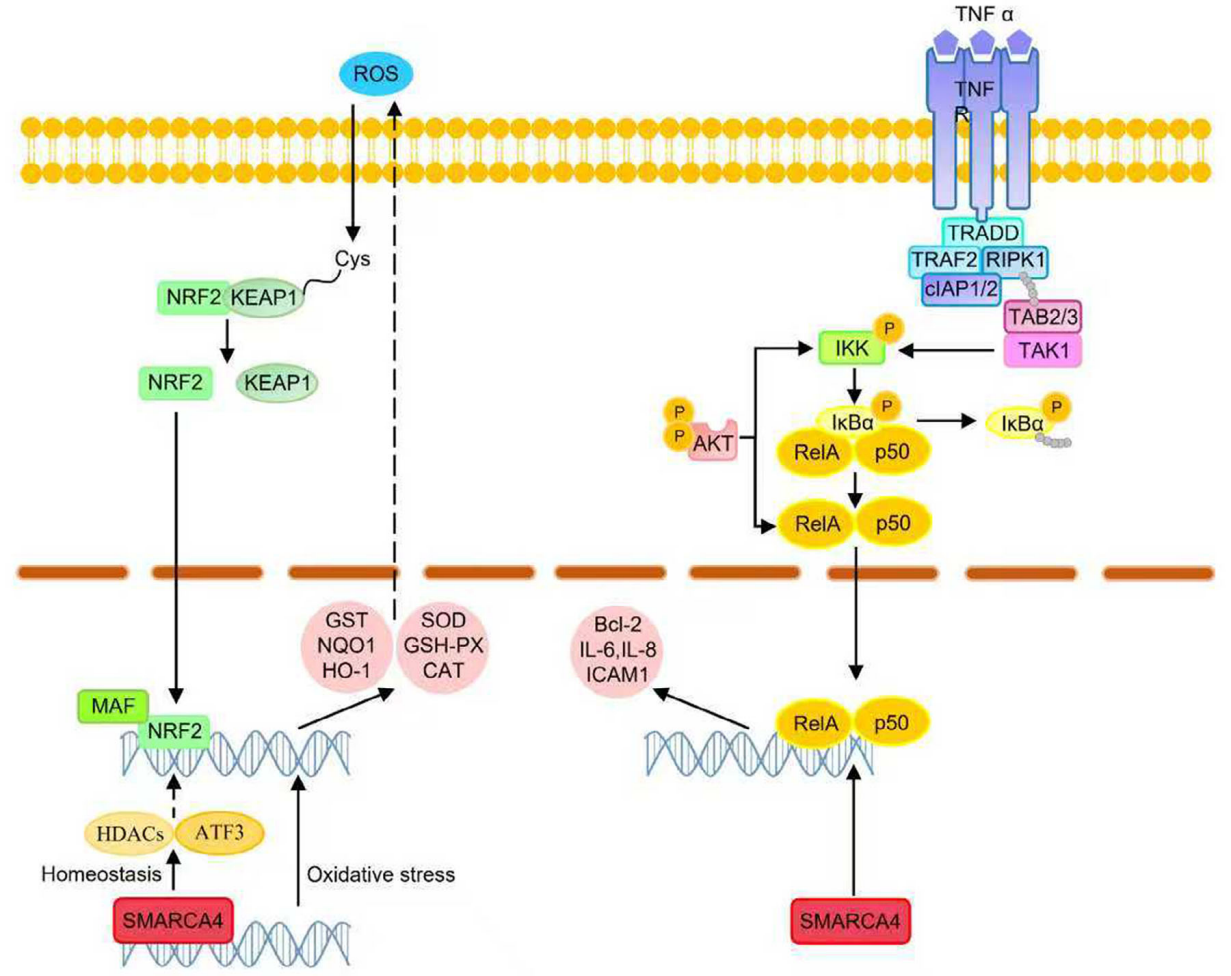
SMARCA4 orchestrates critical signaling cascades in the NRF2 and TNFα/NFκB pathway. Under homeostasis conditions, SMARCA4 constrains excessive NRF2 activation to preserve cellular redox balance. During oxidative stress, SMARCA4 displays dysregulated control over NRF2 via chromatin remodeling, an effect heightened in malignancies. For the TNFα/NFκB pathway, NFκB exerts distinct effects in the early and late stages of activity displays stage‐specific duality (early vs. late) in NSCLC progression, linked to SMARCA4‐mediated chromatin remodeling.

The normal function of SMARCA4 relies on precise regulation by multiple signaling pathways, including NRF2, PI3K/AKT, RA, Wnt/β‐catenin and TNFα/NFκB, which together form an intricate, interdependent network. Dysregulation of these pathways, combined with *SMARCA4* genetic mutations, further compromises SMARCA4 function in NSCLC characterized by SMARCA4 deficiency. Of note, signaling crosstalk occurs among the RA, Wnt/β‑catenin, and PI3K/AKT pathways (**Figure** **3**), while AKT also acts as a positive regulator within the TNFα/NF‑κB signaling cascade (**Figure** **4**). These interconnected networks precisely control gene expression through chromatin remodeling and protein–protein interactions, thereby guiding cell fate decisions.

#### 4.3.1 NRF2 Signaling Pathway

The NRF2 signaling pathway maintains cellular redox homeostasis and enhances antioxidant defenses by controlling gene expression. Under basal conditions, NRF2 predominantly resides in the cytoplasm, where it is anchored to the Kelch domain of KEAP1 via conserved ETGE and DLG motifs, forming a two‐site “anchor‐and‐zipper” configuration [71]. This interaction allows the Cullin 3‐RING‐box protein 1‐KEAP1 (Cul3‐Rbx1‐KEAP1) E3 ubiquitin ligase complex to target NRF2 for proteasomal degradation, thereby restricting its cytoplasmic accumulation and preventing aberrant activation of antioxidant and detoxification programs [72, 73]. Under oxidative stress induced by reactive oxygen species (ROS), pharmaceuticals, or electrophilic agents, cysteine modifications in KEAP1 trigger conformational changes that reduce its affinity for NRF2 [74]. Concurrently, kinases such as PI3K and MAPKs phosphorylate NRF2 to modulate its activity [75, 76]. Upon dissociation from KEAP1, stabilized NRF2 exposes its NLS and undergoes importin‐α/β‐mediated nuclear translocation [77, 78]. In the nucleus, NRF2 forms heterodimers with small musculoaponeurotic fibrosarcoma (MAF) proteins (e.g., MAF‐G/K/F) through its bZIP domain; these heterodimers are essential for ARE recognition [79]. The NRF2‐MAF complexes then recruit transcriptional co‐activators such as CBP/p300, which possess intrinsic histone acetyltransferase (HAT) activity. These co‐activators acetylate histones at the target gene promoter, relaxing chromatin structure to facilitate the recruitment of RNA polymerase II and the assembly of the transcription initiation complex [80].

NRF2 target genes encode key antioxidant enzymes, including SOD, GSH‐PX, and CAT, as well as phase II detoxification enzymes such as GST, NQO1, and HO‐1 [81]. Specifically, superoxide dismutase (SOD) catalyzes the dismutation of superoxide anion into hydrogen peroxide and oxygen; glutathione peroxidase (GSH‐PX) reduces peroxides using reduced glutathione (GSH); and catalase (CAT) decomposes hydrogen peroxide into water and oxygen. Collectively, these enzymes work in concert to eliminate intracellular ROS and mitigate oxidative damage [82, 83, 84]. Phase II detoxification enzymes facilitate the removal of harmful substances: glutathione S‐transferase (GST) conjugates electrophiles with glutathione to promote their excretion; NAD(P)H quinones oxidoreductase 1 (NQO1) reduces quinone to hydroquinones, thereby suppressing ROS generation through redox cycling; and heme oxygenase‐1 (HO‐1) catalyzes the degradation of heme into biliverdin, carbon monoxide (CO), and free iron. Biliverdin reductase subsequently converts bilirubin to biliverdin; both biliverdin and bilirubin function as potent antioxidants, whereas CO confers cytoprotection through vasomodulation and anti‐inflammatory effects [85, 86]. Upon resolution of oxidative stress, nuclear NRF2 dissociates from AREs, interacts with KEAP1 or other regulators (e.g., Bax inhibitor‐1, BI‐1), and is exported to the cytoplasm. There, KEAP1‑mediated sequestration of NRF2 restores basal activity and re‑establishes signaling homeostasis.

Physiologically, SMARCA4 functions as a repressor of NRF2 signaling to maintain redox homeostasis; pathologically, aberrant regulation of this pathway promotes tumorigenesis and therapeutic resistance. Under basal conditions, nucleosomal packaging maintains NRF2 target gene promoters in a closed chromatin state, precluding assembly of the transcription initiation complex and thereby suppressing NRF2‐mediated gene expression. SMARCA4 facilitates the binding of AP‐1 transcription factors, such as activating transcription factor 3 (ATF3), which in turn inhibit NRF2‑mediated antioxidant signaling [87]. Specifically, nuclear ATF3 binds the NRF2 promoter and recruits histone deacetylases (HDACs), forming repressor complexes that hinder RNA polymerase II recognition [88]. During acute oxidative stress (still a physiological adaptation), SMARCA4 coordinates chromatin relaxation through two mechanisms: (1) modulation of repressors to alleviate promoter silencing, and (2) rapid decompacting chromatin via HDAC involvement [89]. This remodeling permits NRF2 nuclear entry, ARE engagement, and transcriptional activation. Attenuated NRF2 activity, as demonstrated by reduced expression of the ARE‑driven gene solute carrier family 7 member 11 (SLC7A11), sensitizes cells to ferroptosis under oxidative stress [90]. In cancer cells, loss of SMARCA4 function triggers a metabolic switch toward glycolysis, resulting in increased ROS production [91]. Although moderate ROS levels typically stimulate protective NRF2 signaling, sustained chromatin abnormalities caused by SMARCA4 deficiency disrupt NRF2‐ARE binding, leaving the antioxidant response functionally compromised despite stress‐induced NRF2 upregulation. Uncontrolled ROS accumulation then inflicts oxidative damage on macromolecules and activates oncogenic pathways, thereby driving malignant proliferation and invasion [92].

#### 4.3.2 PI3K/AKT Signaling Pathway

Ligands such as EGF and insulin‐like growth factor (IGF) engage their cognate RTKs (e.g., EGFR, IGFR) on the cell membrane [93]. Ligand binding triggers receptor dimerization and activation of intrinsic tyrosine kinase activity, resulting in receptor autophosphorylation on specific tyrosine residues. These phosphotyrosine motifs act as docking sites for downstream adaptor proteins, which recruit PI3K to the membrane and promote its activation [94]. Activated PI3K phosphorylates phosphatidylinositol 4, 5‐bisphosphate (PIP2) to generate phosphatidylinositol 3, 4, 5‐trisphosphate (PIP3). The accumulation of PIP3 generates a negatively charged membrane microdomain that facilitates its association with the pleckstrin homology (PH) domain of AKT, thereby recruiting cytosolic AKT to the plasma membrane. At the membrane, phosphoinositide‐dependent kinase 1 (PDK1) phosphorylates and activates AKT [95, 96]. Once activated, AKT phosphorylates a range of downstream effectors: it stimulates mammalian target of rapamycin complex 1 (mTORC1), either directly or indirectly through inhibitory phosphorylation of tuberous sclerosis complex 2 (TSC2), thereby promoting cell growth and survival [97]. It phosphorylates and inhibits forkhead box O (FOXO) transcription factors, leading to their cytoplasmic retention, upregulation of BCL2, and suppression of apoptosis [98]. It inactivates GSK‐3β, which enhances glycolysis and metabolic activity [99], and phosphorylates and activates mouse double minute 2 homolog (MDM2), promoting p53 degradation and blocking p53‐mediated apoptosis [100]. Collectively, these AKT‐driven processes critically support tumor cell proliferation and metastasis. Negative feedback mechanisms restrain pathway hyperactivity: mTORC1 activation both suppresses PI3K activity (reducing PIP3 production) and stimulates phosphatase and tensin homolog (PTEN), which dephosphorylates PIP3 back to PIP2 [101]. This feedback loop attenuates AKT activation and modulates overall pathway output.

SMARCA4 modulates the PI3K/AKT pathway predominantly through its interaction with transcription factor Sex‐determining region Y‐box 4 (SOX4), with which it forms a functional complex. Evidence indicates that SOX4 directly controls transcription of the Transforming growth factor β receptor 2 (TGFBR2) gene [102]. In basal‐like tumors, co‐overexpression of SMARCA4 and SOX4 facilitates the assembly of this complex. Under normal conditions, compact chromatin restricts the accessibility of transcription factors and RNA polymerase II access to target genes. Leveraging its chromatin‑remodeling activity, the SMARCA4‐SOX4 complex opens the regulatory region of TGFBR2, thereby enabling its transcription [103]. Once SOX4 binds to the TGFBR2 promoter and transcription initiation, the newly synthesized TGFBR2 protein potentiates both canonical and noncanonical TGF‐β signaling. Canonical TGF‐β signaling is mediated by TGF‑β ligand engagement of TGFBR2 (type II receptor), which subsequently recruits and phosphorylates TGFBR1 (type I receptor) to form an active receptor complex. In parallel, TGFBR2 directly recruits and activates PI3K, thereby initiating the noncanonical PI3K/AKT pathway. Thus, elevated TGFBR2 expression amplifies TGF‐β‑dependent signaling and downstream PI3K/AKT pathway activation through these two interconnected cascades [104].

Under physiological conditions, the engagement of TGF‐β ligands with TGFBR2 and TGFBR1 triggers the assembly of a receptor complex, leading to conformational changes and TGFBR2‑mediated phosphorylation of TGFBR1 [105]. Activated TGFBR1 subsequently phosphorylates SMAD family member 2 (Smad2) and SMAD family member 3 (Smad3). These phosphorylated Smads associate with SMAD4 to form heteromeric complexes that translocate to the nucleus [106]. Within the nucleus, the SMAD complexes bind TGF‐β response elements (TBEs) in the PTEN promoter. Given that PTEN acts as a major negative regulator of the PI3K/AKT pathway, its transcriptional induction suppresses pathway activity, thereby restraining cell proliferation and survival [107]. Conversely, during tumorigenesis, TGF‐β signaling promotes AKT activation by engaging integrin‐linked kinase (ILK) or through growth factor receptor‐bound protein 2 (Grb2)‐mediated Ras recruitment [108]. Loss of SMARCA4 leads to enrichment of histone H3K27me3 at the PTEN promoter. This combined PTEN inactivation and SMARCA4 deficiency creates a synergistic “double hit” that induces synthetic lethality and drives hyperactivation of the PI3K/AKT pathway [109, 110]. Sustained pathway activation subsequently stimulates mTOR‐dependent cell cycle regulators, thereby accelerating cell‐cycle progression, enhancing glycolysis, and promoting invasion and metastasis. Additionally, dysregulated PI3K/AKT signaling heightens genomic instability, further fueling tumor initiation and progression.

#### 4.3.3 Retinoic Acid (RA) Signaling Pathway

The RA signaling pathway, essential for the metabolism of vitamin A (retinol; ROL), is critically involved in the regulation of cell differentiation, proliferation, and development. In the systemic circulation, retinol is transported in a stable complex with retinol‐binding protein (RBP) [111]. Cellular uptake of retinol occurs through the binding of this RBP‐retinol complex to specific cell‐surface transporters, most notably stimulated by retinoic acid 6 (STRA6) [112]. Once internalized, retinol is enzymatically oxidized to RA, which initiates the signaling cascade. Subsequently, RA is shuttled to the nucleus by cytoplasmic retinoic acid‐binding proteins (CRABPs). Within the nucleus, RA binds to retinoic acid receptors (RARs) and retinoid X receptors (RXRs), driving the formation of RAR/RXR heterodimers [113]. These heterodimers act as the central platform of transcriptional regulatory complexes: they recognize retinoic acid response elements (RAREs) located in target gene promoters and recruit transcriptional coregulators (coactivators or corepressors), thereby modulating gene expression to control cellular proliferation and differentiation [114].

In NSCLC, the RARβ gene frequently undergoes promoter hypermethylation [115]. This epigenetic silencing compromises RA‐mediated growth suppression and differentiation, thereby contributing to tumor development. Concurrently, RA deficiency accelerates tumor progression through two mechanisms: loss of the ability to suppress β‐catenin nuclear translocation and enhances the activation of the transcriptional coactivators Yes‐associated protein (YAP) and transcriptional coactivator with PDZ‐binding motif (TAZ) [116, 117].

SMARCA4 modulates RA signaling primarily by remodeling chromatin and facilitating RAR/RXR complexes‑mediated transcriptional activation. This regulation depends on the recognition of acetylated lysine residues on RAR/RXR complex components by SMARCA4's bromodomain. Upon RA pathway activation, SMARCA4 is recruited to RAR/RXR complexes in a bromodomain‑dependent manner. Its ATP‐dependent chromatin remodeling activity subsequently relaxes chromatin at target promoter regions (e.g., CYP26A1), enabling the assembly of RNA polymerase II and basal transcription factors to initiate transcription [118, 119]. Collectively, these actions promote oncogenesis: by remodeling chromatin and upregulating CYP26A1, SMARCA4 accelerates RA catabolism, thereby counteracting the tumor‐suppressive functions of RA.

#### 4.3.4 Wingless‐Related Integration Site/β‐Catenin (Wnt/β‐Catenin) Signaling Pathway

The Wnt/β‐catenin pathway regulates cell proliferation and differentiation by controlling the stability and nuclear function of β‑catenin [120]. Pathway activation is initiated when Wnt ligands engage Frizzled receptors and recruit LRP5/6 co‐receptors to form a receptor complex [121]. This assembly exposes LRP5/6 phosphorylation sites, leading to the activation of Dishevelled (Dvl) [122]. In the absence of Wnt signaling, a destruction complex sequentially phosphorylates β‐catenin via casein kinase 1 (CK1) and GSK‐3β, targeting it for β‐transducin repeat‐containing protein (β‐TrCP)‐mediated ubiquitination and subsequent proteasomal degradation [123]. Activated Dvl suppresses this degradation, thereby permitting the cytoplasmic accumulation of β‐catenin. Stabilized β‐catenin then translocates to the nucleus, where it interacts with T‐cell factor/lymphoid enhancer factor (TCF/LEF) transcription factors, and activates target genes (e.g., c‐Myc, Cyclin D1) that promote cell survival and proliferation. Emerging evidence also links the destruction complex to the regulation of YAP/TAZ signaling, revealing additional roles in mechanotransduction [124].

Dysregulated β‐catenin expression is commonly observed in NSCLC and leads to elevated c‐Myc levels. c‐Myc drives tumor progression through multiple mechanisms, including (1) accelerating the G1/S phase transition by upregulating CDK4/6 and Cyclin D1; (2) suppressing apoptosis by downregulating pro‐apoptotic proteins such as BIM and PUMA; and (3) enhancing glycolysis and glutamine metabolism to sustain proliferative demands [125, 126, 127, 128, 129]. Clinically, MYC amplification acts as an independent predictor of poor prognosis, and high c‐Myc expression is associated with markedly shorter OS in NSCLC patients [130].

SMARCA4 interacts with β‐catenin through its bromodomain, which recognizes acetylated lysine residues and thereby potentiates β‐catenin's transcriptional activity. Functioning as a transcriptional coactivator, it directly associates with β‐catenin‐TCF/LEF complexes [131, 132]. Its ATP‐dependent chromatin remodeling activity increases the accessibility of transcription factors and RNA polymerase II at the promoters of Wnt target genes [133]. By mediating histone contacts and protein–protein interactions, SMARCA4 couples chromatin remodeling to transcriptional regulation, thereby serving as a critical regulatory hub in the Wnt/β‐catenin pathway. Consequently, loss or mutational inactivation of SMARCA4 disrupts this regulatory axis, driving aberrant Wnt signaling and tumorigenesis.

#### 4.3.5 Tumor Necrosis Factor α/Nuclear Factor kappa B (TNFα/NFκB) Signaling Pathway

The TNFα/NFκB pathway, which is activated through tumor necrosis factor receptors (TNFR), regulates inflammation, cell survival, and apoptosis [134]. Signaling transduction is initiated upon binding of TNF (secreted by immune cells) to TNFR, triggering receptor trimerization and exposure of the death domain (DD) [135]. This event promotes the recruitment of TNFR1‐associated death domain protein (TRADD), receptor‐interacting protein kinase 1 (RIPK1), and tumor necrosis factor receptor‐associated factors 2/5 (TRAF2/5) to assemble Complex I, which then incorporates cellular inhibitor of apoptosis proteins (cIAP1/2) [136]. Functioning as E3 ubiquitin ligases, cIAP1/2 mediate the K63‐linked polyubiquitination of RIPK1. This modification masks the death domain of RIPK1 to prevent apoptosis, stabilizes Complex I, and facilitates the recruitment of TGF‐β‐activated kinase 1–TAK1‐binding protein 2/3 (TAK1‐TAB2/3) complex. TAB2/3 serves as a bridge connecting TAK1 to Complex I, thereby inducing TAK1 autophosphorylation. Activated TAK1 subsequently phosphorylates IKKβ, leading to full activation of the IKK complex [137]. The activated IKK complex phosphorylates IκBα, marking it for K48‐linked ubiquitination and subsequent proteasomal degradation. This releases NFκB dimers (e.g., p65/RelA‐p50) and exposes their nuclear localization signals (NLS), allowing importin‐α/β‐mediated translocation into the nucleus [138]. Once in the nucleus, NFκB drives the transcription of anti‐apoptotic genes (e.g., Bcl‐2), pro‐inflammatory (e.g., IL‐6, IL‐8), and pro‐survival genes (e.g., c‐IAP2) [139]. However, sustained TNF signaling can also activate pro‐apoptotic pathways through TRADD‐dependent caspase cascades, ultimately inducing apoptosis [140].

In early‐stage NSCLC, NFκB induces cell cycle arrest to suppress tumor progression [141]. Conversely, in advanced disease, NFκB promotes tumor growth by enhancing survival (through Bcl‐2 upregulation and caspase inhibition) and stimulating angiogenesis (via VEGF induction) [142]. In EGFR‐mutant NSCLC, EGFR hyperactivation augments NFκB activity through the PI3K/AKT pathway: (1) AKT directly phosphorylates IκB inhibitors to activate NFκB, (2) AKT inhibits GSK‐3β–dependent β‐catenin degradation, and (3) AKT phosphorylates the p65 subunit [143, 144, 145]. Collectively, these events potentiate the expression of pro‐survival genes. Upon TNFα stimulation, SMARCA4 regulates gene expression, thereby influencing cell fate and remodeling the tumor microenvironment (TME). Through chromatin relaxation, it facilitates the binding of p65 to its target genes. Loss of SMARCA4 L attenuates NFκB activity, diminishing the production of inflammatory cytokines (e.g., IL‐6), reducing PD‐L1 expression, impairing T‐cell infiltration, and fostering immune escape [146, 147].

### 4.4 SMARCA4 Deficiency and EGFR Pathway Activation Drive Osimertinib Resistance in EGFR‐Mutant NSCLC

Although the co‑occurrence of SMARCA4 deficiency and *EGFR* mutations is relatively rare in NSCLC, this deficiency cooperates with heightened EGFR signaling and compensatory upregulation of SMARCA2 (BRM) to promote osimertinib resistance.

#### 4.4.1 Osimertinib Resistance Mediated by SMARCA4/NFκB‐Driven YAP1‐AXL Axis

Activation of the YAP1‐AXL signaling hub integrates inputs such as integrin engagement, epithelial cadherin (E‐cadherin)‐mediated adhesion, cytoskeletal dynamics, and NFκB to regulate transcription [148, 149]. Canonical Hippo pathway activation begins when Salvador homolog 1 (Sav1) facilitates mammalian sterile 20‐like kinase 1/2 (MST1/2)‐dependent phosphorylation of large tumor suppressor kinase 1/2 (LATS1/2). Subsequently, LATS1/2, in complex with Mps one binder 1 (MOB1), phosphorylates YAP and TAZ [150, 151, 152]. This phosphorylation event promotes 14‐3‐3 protein binding and sequestration in the cytoplasm [153]. Owing to their structural homology, YAP1 and TAZ form dimers via WW domain interactions [154]. Dephosphorylation by protein phosphatase 2A (PP2A) unmasks the nuclear localization signals (NLS) of YAP/TAZ, enabling importin‐α/β‐mediated nuclear entry [155]. Once in the nucleus, YAP/TAZ associates with TEAD transcription factors to induce expression of CCND1, MYC, and MMP14, thereby driving G1/S phase progression, epithelial‐mesenchymal transition (EMT), and tumorigenesis [156]. Furthermore, YAP1 interacts with β‐catenin to influence TCF/LEF transcriptional activity [157].

**FIGURE 5:**
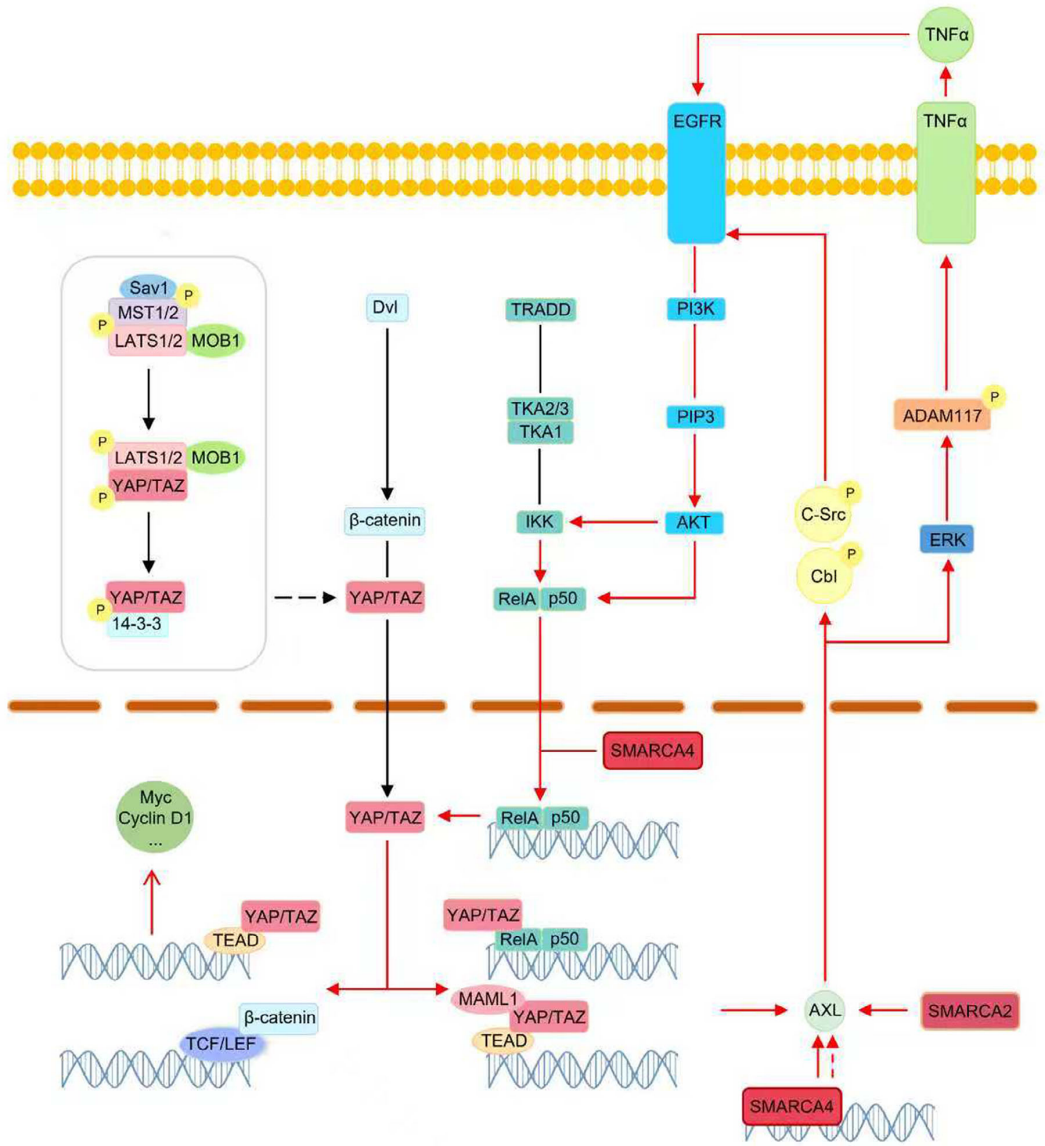
SMARCA4's role in driving drug resistance via the YAP1‐AXL axis through NFκB recognition. SMARCA4 transcriptionally induces YAP1. YAP1 binds to the TEAD motif in the AXL promoter, activating NF‐κB, while NF‐κB indirectly elevates AXL via PI3K/AKT signaling, growth factors, and cytokines, establishing a dual regulatory circuit. In EGFR‐mutant NSCLC, EGFR autophosphorylation activates NF‐κB, which enhances YAP1‐TEAD binding affinity and upregulates AXL. This cascade activates EGFR/PI3K/AKT/MAPK signaling and suppresses EGFR ubiquitination, forming an EGFR→NF→κB→YAP1→AXL positive feedback loop that confers EGFR‐TKI resistance. AXL additionally promotes TGF‐α shedding to paracrinely activate neighboring tumor cells, enabling collective resistance.

SMARCA4 functions as a transcriptional co‑activator by interacting with the NFκB p65 subunit through its bromodomain, thereby establishing an active chromatin state at the YAP1 promoter to drive YAP1 transcription. Subsequently, YAP1 directly occupies TEAD sites within the AXL promoter and associates with p65. NFκB activation upregulates AXL via PI3K/AKT signaling, growth factors (IGF‐1, VEGF), and cytokines (IL‐6, TNF‐α), forming a dual‐regulatory loop [158]. In EGFR‐mutant NSCLC, EGFR autophosphorylation activates NFκB through IKK and RelA. Although LATS1/2‑mediated phosphorylation suppresses YAP1‐TEAD interaction, NFκB facilitates YAP1 dephosphorylation, increasing YAP1‐TEAD binding affinity and recruiting coactivators (e.g., mastermind‐like protein 1, MAML1) to boost AXL expression. Enhanced YAP1 occupancy at the AXL promoter is associated with higher AXL protein levels. Functioning as an RTK, AXL phosphorylates c‐Src and Cbl, stimulating EGFR/PI3K/AKT/MAPK cascades while concurrently blocking EGFR ubiquitination. This creates an EGFR→NFκB→YAP1→AXL→EGFR positive feedback loop that drives TKI resistance [159, 160]. Moreover, AXL indirectly activates EGFR on neighboring cells through TGF‐α shedding mediated by ERK1/2‑phosphorylated a disintegrin and metalloproteinase 17 (ADAM17), thereby facilitating collective resistance [161] (**Figure** **5**).

#### 4.4.2 SMARCA4‐Dependent NRF2 Activation via Chromatin Remodeling Underlies Osimertinib Resistance

Resistance in *EGFR*‐mutant NSCLC is commonly attributed to genetic alterations, such as mutations in the drug target or alterations in other pathway genes. Conversely, SMARCA4‐containing SWI/SNF complexes drive resistance through epigenetic mechanisms, remodeling chromatin and reprogramming gene expression. This epigenetic rewiring circumvents the requirement for coding mutations while simultaneously promoting tumor cell survival, proliferation, and EMT [162].

**FIGURE 6:**
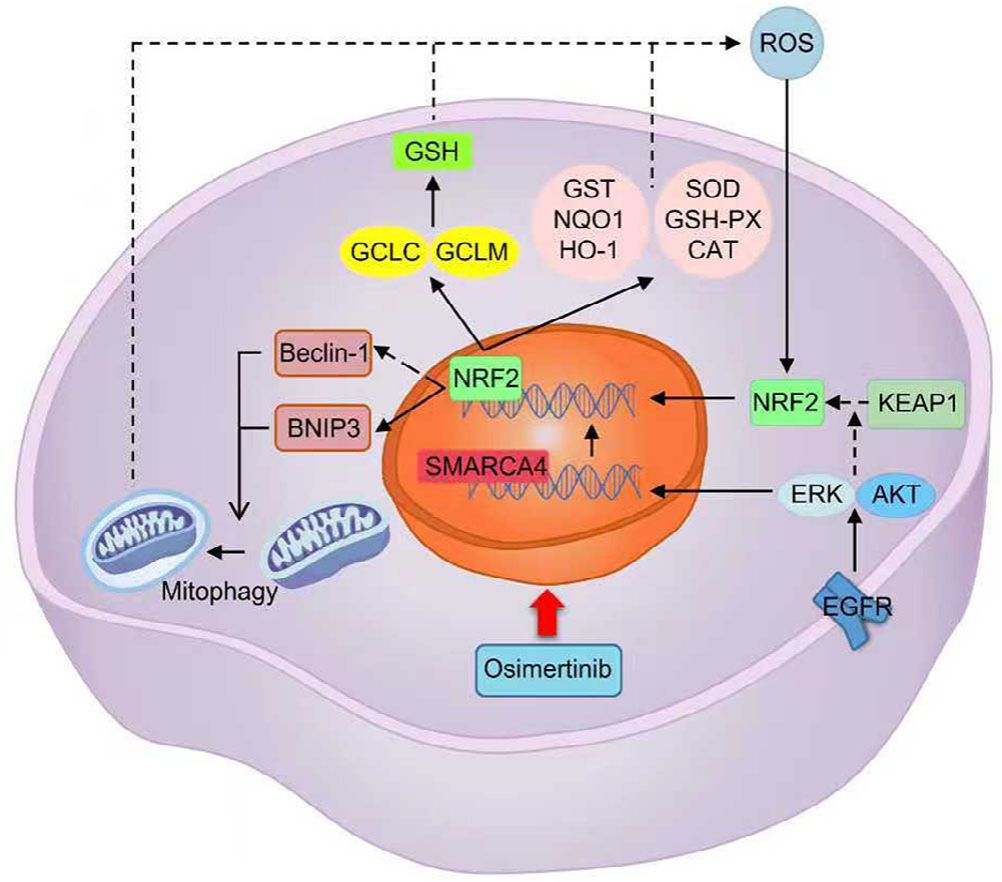
SMARCA4 drives osimertinib resistance by activating the NRF2 signaling pathway, which sustains cytoprotective ROS levels. Osimertinib induces cancer cell death through ROS‐dependent DNA damage. In EGFR‐mutant NSCLC, persistent EGFR autophosphorylation chronically activates PI3K/AKT and MAPK signaling, leading to NRF2 phosphorylation. Concurrently, SMARCA4 induces expression of antioxidant genes via chromatin remodeling. Chronic osimertinib exposure enables NSCLC cells to maintain ROS within a survival‐permissive range through SMARCA4‐enhanced NRF2 activity, fostering therapeutic resistance.

Oxidative stress induces NRF2 overactivation in a manner dependent on the mammalian SWI/SNF (mSWI/SNF) complex, thereby conferring osimertinib resistance. Although osimertinib induces ROS‐mediated DNA damage to exert cytotoxicity, sustained NRF2 activation counteracts ROS accumulation. Prolonged treatment enables adaptive NRF2 upregulation via SWI/SNF, shifting the cellular response from apoptosis toward resistance. SMARCA4 knockdown reverses this by inhibiting proliferation and restoring drug sensitivity [163]. Mechanistically, SMARCA4 remodels chromatin at NRF2‐regulated genes (e.g., *GCLC* and *GCLM*), upregulating antioxidant enzymes that enhance glutathione synthesis and maintain redox balance [164]. Additionally, BRG1/BRM ATPases facilitate ROS clearance via Beclin‐1/BNIP3‐mediated mitophagy [165]. Hypoxic conditions, acting through HIF‐1α‐SWI/SNF interactions, and IL‐6/STAT3 signaling further amplify NRF2 activation, establishing a self‐reinforcing feedback loop that integrates hypoxia, oxidative stress, and antioxidant responses to sustain resistance [166, 167]. KEAP1 plays a critical role in modulating osimertinib resistance. Under physiological conditions, KEAP1 promotes the ubiquitination and subsequent degradation of NRF2. However, more than 10% of NSCLC harbors KEAP1 mutations, frequently located in the BTB domain required for E3 ligase dimerization, thereby impairing NRF2 sequestration and degradation [168]. NRF2 mutations (e.g., in ETGE/DLG motifs/ubiquitination sites) also evade KEAP1‐mediated regulation, leading to constitutive NRF2 activation and antioxidant gene upregulation [169]. In EGFR‐mutant NSCLC, sustained EGFR autophosphorylation drives hyperactivation of the PI3K/AKT/MAPK pathways. Oxidative stress and these kinases in turn phosphorylate NRF2, disrupting KEAP1 binding and enabling NRF2 nuclear translocation [170]. SMARCA4 further amplifies the expression of NRF2 target genes via chromatin remodeling. Taken together, osimertinib‐induced ROS, KEAP1/NRF2 mutations, and EGFR‐driven PI3K/AKT/ERK signaling converge to drive NRF2 hyperactivation. This elevates antioxidants, sustaining ROS at pro‐survival levels and thereby promoting therapeutic resistance (**Figure** **6**).

In summary, the concomitant loss of SMARCA4 and enhanced EGFR signaling drives osimertinib resistance in EGFR‐mutant NSCLC, involving SMARCA4/NFκB‐mediated activation of the EGFR→NFκB→YAP1→AXL positive feedback loop and SMARCA4‐induced NRF2 hyperactivation via chromatin remodeling, which is further amplified by KEAP1/NRF2 mutations and EGFR‐driven signaling cascades.

## 5 EGFR‐Independent Resistance via Bypass Pathway Activation in EGFR‐Mutant NSCLC

Bypass pathway activation is driven by genetic alterations, including mutations in genes downstream of EGFR, gene fusions, gene amplifications, and mutations in genes encoding cell cycle‐related proteins. Representative mechanisms encompass amplification of mesenchymal–epithelial transition (MET) factor, Hepatocyte growth factor (HGF) overexpression, amplification of HER2, activation of HER3, and AXL upregulation.

### 5.1 MET Amplification and HGF overexpression

MET, a member of the RTK family, is frequently amplified and strongly associated with TKI resistance in EGFR‑mutant lung cancers. HGF, the canonical ligand for MET, is predominantly secreted by both lung cancer cells and tumor‑associated stromal cells. Binding of HGF to MET triggers a spectrum of biological responses, including mitogenic, morphogenic, and anti‑apoptotic effects, and reactivates the downstream PI3K/Akt signaling cascade [171, 172, 173]. This reactivated signaling drives TKI resistance and contributes to tumorigenesis, proliferation, and metastasis in EGFR‑mutant NSCLC [174]. Notably, HGF has also been shown to promote MET amplification in vitro and in vivo by enriching pre‑existing MET‑amplified cell clones [175, 176]. Collectively, activation of the MET signaling axis, whether via MET gene amplification or HGF‑dependent ligand stimulation, constitutes a major bypass resistance mechanism that enables *EGFR*‑mutant lung cancer cells to evade TKI therapy. Focal MET amplification drives gefitinib resistance in the *EGFR*‑mutant, gefitinib‑sensitive lung cancer cell line HCC827 by inducing ErbB3 phosphorylation, which reactivates the PI3K/Akt signaling axis and thereby bypasses the inhibitory effects of gefitinib on EGFR. Knockdown of MET using MET‑specific short hairpin RNA (shRNA) restored the ability of gefitinib to suppress the PI3K/Akt pathway, whereas ErbB3‑targeted shRNA inhibited Akt phosphorylation and arrested cell cycle progression in resistant cells [177]. Low‑frequency MET‑amplified clones have been shown to pre‑exist in untreated HCC827 cells and approximately 4% of NSCLC patients, and expand under TKI selective pressure to become dominant clones that mediate clinical resistance to gefitinib or erlotinib [178]. HGF‑induced MET activation represents a distinct, amplification‑independent resistance mechanism; as reported by Yano et al., HGF‑mediated MET signaling reactivates the PI3K/Akt pathway in an ErbB3‑independent manner in *EGFR*‑mutant lung adenocarcinoma, thereby conferring gefitinib resistance [179]. PC‑9 and HCC827 cells, both gefitinib‑sensitive *EGFR*‑mutant lung adenocarcinoma lines, do not secrete detectable HGF under baseline conditions, yet exogenous HGF protected these cells from gefitinib‑induced apoptosis in a dose‑dependent manner [180].

### 5.2 Amplification of HER2 and HER3 activation

Initial preclinical studies demonstrated that gefitinib exerts a prominent antiproliferative effect on tumors with ErbB2 overexpression, giving rise to the traditional notion that increased *ErbB2* gene copy number correlates with greater sensitivity to gefitinib [181, 182]. However, multivariate analysis has confirmed that *EGFR* mutation status is a more powerful predictor of favorable clinical outcomes in gefitinib‐treated NSCLC patients than *ErbB2* or *EGFR* copy numbers [183]. Notably, ErbB2‐mediated TKI resistance is closely intertwined with ErbB3, another key member of the ErbB family. ErbB3 has been identified as a critical mediator of resistance to EGFR‐ or ErbB2‐directed TKIs across multiple malignancies [184, 185]. As a unique inactive kinase, ErbB3 cannot exert its biological effects independently; instead, it r depends on transactivation and transphosphorylation through heterodimerization with other ErbB family members, particularly ErbB2 [186]. Functionally, ErbB3 plays a compensatory role by substituting for TKI‐inhibited EGFR or ErbB2 to initiate and sustain PI3K/Akt signaling activation. When ErbB2 is inhibited by TKIs, upregulation of ErbB3 expression and reduced ErbB3 phosphatase activity attenuate the inhibitory effects of TKIs on ErbB2, resulting in gefitinib and erlotinib resistance [187]. Distinct from EGFR and ErbB2, activating PI3K via adaptor proteins, ErbB3 directly interacts with the p85 regulatory subunit of PI3K, highlighting the prominent role of ErbB3‐driven resistance in TKI‐treated tumors [188].

### 5.3 AXL Upregulation and Crosstalk With SMARCA4

AXL is one of three RTKs in the TAM family, which also includes TYRO3 and MERTK. These three receptors share two secreted ligands, protein S (PROS1) and growth‐arrest‐specific 6 (GAS6), both of which exhibit structural and functional homology [189, 190]. Structurally, TAM family members contain three conserved domains: two extracellular domains (immunoglobulin‐like and fibronectin type III domains) and one intracellular kinase domain. Through ligand‐induced dimerization and kinase activation, TAM receptors transduce extracellular signals into intracellular signaling responses that govern biological processes such as inflammation and phagocytosis [191]. In *EGFR*‐mutant NSCLC, AXL functions as a key mediator of acquired resistance to multiple EGFR‐TKIs, including erlotinib, gefitinib, afatinib, and osimertinib [192, 193]. Mechanistically, AXL cooperates with EGFR and HER3 to promote cellular tolerance to Osimertinib, and signals downstream through the phospholipase Cγ (PLCγ)‐protein kinase C (PKC) axis to upregulate mTOR, a critical effector of the PI3K pathway [194]. AXL also contributes to drug resistance via autophagy‐dependent mechanisms and is upregulated in the drug‐tolerant persister (DTP) state upon treatment with EGFR‐TKIs. Moreover, AXL overexpression is tightly linked to epithelial‐mesenchymal transition (EMT), a core phenotype of TKI resistance, and paracrine crosstalk between cancer cells and cancer‐associated fibroblasts (CAFs) elevates GAS6 secretion, further enhancing AXL activation and EMT [195, 196]. Additionally, AXL engages in crosstalk with other oncogenic drivers, such as KRAS and YAP1, acting as both a target and a regulator of YAP1 to form a feedback amplification loop that reinforces oncogenic signaling [197, 198].

EGFR mutations drive YAP1 hyperactivation via a self‐reinforcing Hippo‐YAP feedback loop [159, 160]. In a functionally distinct regulatory axis, SMARCA4 inversely correlates with YAP1 activity and represses AXL expression. Thus, SMARCA4 deficiency relieves this repression, sustains the EGFR–YAP1–AXL feedback cycle, and facilitates YAP1 nuclear localization [199]. Truncating mutations in Class I SMARCA4 are strongly associated with reduced OS in NSCLC [146]. Following SMARCA4 depletion, SMARCA2 (which shares 75% homology with SMARCA4) assembles noncanonical SWI/SNF (ncBAF) complexes with SMARCC1/ARID1A. Though its ATPase activity is 2.3‐fold higher, SMARCA2 displays reduced precision at enhancer [200]. While functioning as a tumor‐suppressive in *SMARCA4* wild‐type settings, SMARCA2 acquires oncogenic properties in SMARCA4‐deficient tumors through synthetic lethality. Subsequent enhancer remodeling by SMARCA2 upregulates YAP/TEAD‐ target genes (e.g., CTGF, CYR61), driving tumor cell persistence and TKI resistance [201].

Collectively, bypass pathway activation constitutes a critical mechanism of EGFR‑independent resistance in EGFR‑mutant NSCLC, driven primarily by MET amplification and HGF overexpression, HER2 amplification coupled with compensatory HER3 activation, and AXL upregulation. These alterations reactivate downstream oncogenic cascades such as PI3K/AKT and EGFR‐YAP1‐AXL axis, thereby aggravating TKI resistance and offering pivotal targets for overcoming acquired resistance to EGFR‑TKIs.

## 6 Targeted Therapies against Resistance‐Related Molecules/Pathways

Despite the remarkable clinical efficacy of osimertinib in EGFR‑mutant NSCLC, acquired resistance persists as a formidable challenge, mainly attributable to EGFR‑dependent mutations, SMARCA4‑related epigenetic reprogramming, and activation of bypass signaling pathways. Consequently, therapeutic strategies that specifically targeted these resistance‑associated molecules and signaling nodes have emerged as a central strategy to overcome treatment failure.

### 6.1 Targeting EGFR‐Dependent Resistance

EGFR‐dependent resistance represents the predominant cause of treatment failure in *EGFR*‐mutant NSCLC treated with EGFR‐TKIs, largely attributable to the acquisition of secondary *EGFR* mutations. Departing from conventional exon‐based classification, Robichaux et al. proposed a structure‐function‐based classification of *EGFR* mutations to devise tailored strategies for overcoming EGFR‐dependent resistance, and innovatively defined the PACC (P‐loop and αC‐helix compressing) subtype as a functionally distinct category [202].

PACC mutations, which include G719X, S768I, and C797S among other mutations predicted to reorient the P‐loop or αC‐helix, cause resistance to third‐generation TKIs such as osimertinib. By contrast, second‐generation TKIs display significantly higher selectivity and efficacy because they avoid interaction with the EGFR P‐loop and sustain stable binding within the hydrophobic cleft [202]. The remaining subgroups consist of classical‐like mutations (distant from the ATP‐binding pocket), T790M‐like mutations (in the hydrophobic core), and Ex20ins‐L (insertions in the loop at the C‐terminal end of the αC‐helix in exon 20). T790M‐like mutations are further subdivided into third‐generation TKI‐sensitive (T790M‐like‐3S) and resistant (T790M‐like‐3R) subtypes. The latter encompasses complex mutations in which T790M co‐occurs with additional drug‐resistant mutations such as C797S, L718X, and L792H; T790M‐like‐3S mutants remain responsive to third‐generation TKIs, whereas T790M‐like‐3R mutants retain selectivity to ALK/PKC inhibitors (e.g., brigatinib or midostaurin) [203, 204, 205, 206, 207]. EGFR exon 20 insertion mutations exhibit a heterogeneous TKI sensitivity, prompting further division of Ex20ins‐L into near‐loop (Ex20ins‐NL) and far‐loop (Ex20ins‐FL) subtypes [202, 208]. Ex20ins‐NL mutants display greater sensitivity to second‐generation TKIs and Ex20ins‐active inhibitors, while Ex20ins‐FL mutants display relatively low sensitivity [209]. Although classical‐like mutations are generally sensitive to all classes of EGFR TKIs, structure‐function classification enables a more precise prediction of resistance patterns when these mutations co‐occur with other EGFR mutations.

EGFR Ex20ins insertion mutations (Ex20ins) represent the third most prevalent subtype (approximately 6%–12%) of EGFR alterations, following ex19 del and L858R [210]. While the vast majority of these patients exhibit intrinsic resistance to first and second‐generation EGFR‐TKIs. Platinum‐based chemotherapy is the standard first‐line treatment for this patient cohort, but treatment outcomes remain suboptimal, highlighting a significant unmet medical need for novel therapeutic strategies in patients with EGFR ex20ins [211, 212]. Minchom et al. [213] reported results from the CHRYSALIS phase I/II open‑label trial, demonstrating that in postplatinum patients with EGFR ex20ins‑mutant advanced NSCLC, amivantamab was associated with improved outcomes compared with external controls, including a 10‑month extension in OS, an ORR of 40%, a median PFS of 8.3months, a median time to next therapy (TTNT) of 14.8 months, and a median OS of 22.8 months. Mobocertinib also yielded substantially better outcomes (ORR 35.1%, median PFS 7.3 months, and median OS 24 months) relative to a real‑world data (RWD) cohort of platinum‐pretreated patients receiving conventional therapies (ORR 11.9%, median PFS 3.3 months, and median OS 11.5 months) [214]. Both mobocertinib and amivantamab showed comparable efficacy in patients with *EGFR* ex20ins‑mutant NSCLC whose disease progressed during or after platinum‑based chemotherapy [215]. The phase III open‑label EXCLAIM‑2 trial compared mobocertinib with platinum‑based chemotherapy as first‑line treatment for EGFR ex20ins‑positive advanced/metastatic NSCLC; however, the trial did not meet its primary endpoint, as mobocertinib failed to demonstrate superiority over platinum‑based chemotherapy in this first‑line setting [216]. Furthermore, the phase I/II open‑label REZILIENT1 trial demonstrated that zipalertinib achieved clinically meaningful efficacy in patients with EGFR ex20ins‑mutant NSCLC previously treated with platinum‑based chemotherapy, with or without prior amivantamab, yielding a confirmed ORR of 35.2% and median duration of response (DOR) of 8.8 months [217].

### 6.2 Targeting SMARCA4‐Related Resistance

SMARCA4, the catalytic SWI/SNF ATPase complex that regulates gene expression and DNA repair, is frequently deficient in EGFR‑mutant NSCLC; its loss dysregulates EGFR signaling and drives osimertinib resistance [218]. Immune checkpoint inhibitors (ICIs) have emerged as one of the core therapeutic modalities for NSCLC. High TMB is believed to promote neoantigen generation and enhance tumor immunogenicity, thereby improving immunotherapeutic responses; similarly, elevated programmed death ligand 1 (PD‐L1) expression has been consistently linked to greater ICI sensitivity. In *EGFR*‐mutant NSCLC, however, SMARCA4 deficiency gives rise to heterogeneous immunological phenotypes, substantially complicating the prediction of immunotherapy benefit [219, 220]. SMARCA4 deficiency fosters an immunosuppressive tumor microenvironment characterized by reduced dendritic cells and CD4^+^ T cells, along with increased FOXP3^+^ regulatory T cells and CD66b^+^ neutrophils [221, 222]. Although these tumors frequently display low or absent PD‐L1 expression, clinical benefits from ICIs have nonetheless been observed in several studies [223]. In ICI‑treated NSCLC cohorts, *SMARCA4* mutations have been associated with improved prognosis in select patient populations. Nevertheless, subgroup analyses of immunotherapy trials revealed that *SMARCA4* class I and class II mutations had no significant effect on patient PFS or OS, whereas patients with homozygous truncating mutations were associated with significantly shorter OS following immunotherapy compared with wild‐type status [224, 225]. Furthermore, analysis of the Foundation Medicine database demonstrated that NSCLC patients harboring *SMARCA4* truncating mutations exhibit elevated TMB levels and a relatively high PD‐L1 positivity rate (15%) [226]. In contrast, co‐occurring mutations in *KRAS*, *STK11*, or *KEAP1* further impair the efficacy of immunotherapy in SMARCA4‑mutant NSCLC [227, 228]. Collectively, these features contribute to an immune‐excluded phenotype that not only fails to achieve the favorable outcomes predicted by PD‐L1 and TMB status but also heightens the risk of hyperprogressive disease during ICI treatment.

Though osimertinib effectively controls EGFR‐mutant NSCLC and reduces CNS metastasis, its activity is limited in SMARCA4‐deficient or resistant tumors. Precision combination strategies are therefore essential for NSCLC harboring concurrent EGFR/SMARCA4 mutations or acquired osimertinib resistance. Therapeutic approaches are tailored according to SMARCA4 status: In NSCLC with wild‐type SMARCA4‐WT and EGFR mutations, enhancing oxidative stress via NRF2 inhibitors (e.g., ML385) or blockade of glutathione synthesis (e.g., Epalrestat) has been validated in both cellular assays and mouse models to promote lung cancer cell death and restore sensitivity of resistant cell lines to EGFR‐TKIs [229, 230]. In NSCLC with concurrent SMARCA4 and EGFR mutations, disruption of the EGFR‐NFκB‐YAP1‐AXL feedback loop through PROTAC‐mediated SMARCA2 degradation (e.g., A947) has shown enhanced growth inhibition in SMARCA4‐mutant cancer models both in vitro and in vivo [231]. Notably, TEAD inhibitors (e.g., K‐975) and adagrasib exert a dual inhibitory effect in *KRAS* G12C‐mutant NSCLC, suggesting that YAP1‐TEAD interference may reverse drug resistance in *EGFR*‐mutant NSCLC [232, 233]. Moreover, preclinical restoration of chromatin remodeling with HDAC inhibitors, the clinical‐stage SMARCA2‐selective degrader PRT3789 (currently in phase I/II clinical trials), and CRISPR‐Cas9 gene editing in C57BL/6J mouse and BALB/c nude mouse models of NSCLC have all demonstrated antitumor activity, suggesting viable resistance strategies to overcome resistance [234, 235, 236].

The crosstalk between SMARCA4 and EGFR represents a key epigenetic mechanism underlying osimertinib resistance in EGFR‐mutant NSCLC. In a panel of osimertinib‐resistant cell lines, including PC9‐OR, YU‐005C, H1975‐OR, and HCC827‐OR, SMARCA4 knockdown shows the most pronounced antiproliferative effect on PC9‐OR cells in the presence of osimertinib, abolishing cell proliferation and reducing the osimertinib EC50 by approximately eightfold. Moreover, SMARCA4 depletion altered approximately 20% of resistance‐associated differentially expressed genes that were enriched in cell proliferation‐related pathways, and reduced chromatin accessibility and transcription of core genes, including AP‐1 family members (FOS, JUNB), BCL2, and CYP26A1. These observations underscore the supportive role of mSWI/SNF complexes in TKI resistance and suggest the potential therapeutic application of mSWI/SNF inhibitors in TKI‐resistant lung cancers that lack SMARCA4 deficiency [163].

### 6.3 Targeting Bypass Pathways

In the setting of acquired MET‐driven resistance following EGFR TKI therapy, preclinical studies have demonstrated the critical importance of dual EGFR and MET inhibition in overcoming this resistance mechanism. For instance, the combination of osimertinib and the MET inhibitor savolitinib exhibits significant antitumor activity [237]. Furthermore, amivantamab, an anti‐EGFR/c‐MET bispecific antibody, simultaneously binds to both EGFR and MET receptors, blocking ligand binding and promoting receptor downregulation. The combination of amivantamab and lazertinib achieves an improved objective response rate (ORR 36%, 95% CI: 22–51) in patients whose osimertinib resistance was driven by EGFR and/or MET [238]. Although ERBB‑targeted TKIs have been approved for HER2‑ or HER3‑driven malignancies, their utility in HER2‑mutant NSCLC is limited by dose‐limiting toxicities or insufficient efficacy. Recent studies demonstrate that zongertinib (BI 1810631), a covalent HER2 inhibitor, potently and selectively blocks HER2 while sparing EGFR, thereby suppressing the growth of cells dependent on HER2‐driven oncogenic signals. Meanwhile, patritumab deruxtecan (HER3‑DXd), an antibody–drug conjugate targeting HER3, produced a confirmed ORR of 29.8% (95% CI: 23.9–36.2), with antitumor activity observed across a broad range of HER3 expression levels and diverse EGFR‑TKI resistance mechanisms [239, 240]. To address AXL upregulation, a variety of AXL inhibitors have been developed and evaluated in preclinical and clinical studies. Based on their binding mode, these inhibitors are classified into two categories: Type I inhibitors bind to the active DFG‑in conformation of the AXL kinase domain (e.g., ONO‐7475, CB469, PF‐02341066/Crizotinib); Type II inhibitors target the inactive DFG‑out conformation, in which the DFG motif faces away from the active site toward the allosteric pocket (e.g., BMS‐907351/Cabozantinib, RXDX‐106/CEP‐40783, MGCD516/Sitravatinib) [241, 242, 243, 244, 245, 246, 247].

## 7 Conclusion and Future Perspectives

Acquired resistance to osimertinib in *EGFR*‐mutated NSCLC is a complex, multifactorial process arising from the intricate interplay of genetic, epigenetic, and microenvironmental alterations, and can be broadly classified into EGFR‑dependent resistance and EGFR‑independent resistance. Tailoring therapeutic strategies to specific resistance subtypes confers clinical benefit, while novel agents under development provide additional options for clinical therapy (**Table** **2**). In the context of EGFR‑dependent resistance, the T790M gatekeeper mutation in EGFR exon 20 accounts for approximately 50% of acquired resistance to first‑ and second‑generation EGFR‑TKIs [6]. Concurrently, secondary mutations within the EGFR tyrosine kinase domain (most notably C797S) and *EGFR* gene amplification represent the core molecular drivers; the emergence of complex compound mutations further diversifies resistance phenotypes. EGFR‑independent resistance is predominantly driven by bypass signaling pathway activation, with MET amplification being the most common mechanism, mediating resistance through PI3K/AKT pathway activation that circumvents EGFR signaling [177].

**TABLE 2:** Recent advances in distinct types of EGFR‐TKI resistance.

Type	Subtype/ Target	Core features	Therapeutic strategy or research advances	References
EGFR‐dependent	Classical‐like	Distant from the ATP‐binding pocket	Sensitive to all classes of EGFR‐TKIs Regimen adjustment based on co‐mutation profile^a^	[202]
	PACC^b^	P‐loop and αC‐helix compressing	Sensitive to second‐generation EGFR‐TKIs Resistant to third‐generation EGFR‐TKIs	[202]
	T790M‐like‐3S^c^	Isolated mutation in the hydrophobic core	Sensitive to third‐generation EGFR‐TKIs Standard option following resistance to other TKIs	[202]
	T790M‐like‐3R^d^	T790M co‐occurring	Resistant to third‐generation EGFR‐TKIs Selectivity for ALK/PKC inhibitors (e.g., brigatinib or midostaurin)	[203, 204, 205, 206, 207]
	Ex20ins‐NL	Ex20ins‐L into near‐loop	Sensitive to second‐generation EGFR‐TKIs Sensitive to Ex20ins‐active inhibitors	[202]
	Ex20ins‐FL	Ex20ins‐L into far‐loop/ Possible alternative treatments for less sensitivity cases	Amivantamab (ORR 40%, PFS 8.3 months, OS 22.8 months)^e^ Mobocertinib (ORR 35.1, PFS 7.3 months, OS 24 months)^f^ RWD (ORR 11.9%, PFS 3.3 months, OS 11.5 months) Mobocertinib was not superior (EXCLAIM‑2)^g^ Zipalertinib (confirmed ORR 35.2%, median DOR 8.8 months)^h^	[213, 214, 215, 216, 217]
SMARCA4‐Related	SMARCA4‐WT	NRF2 pathway overactivation	NRF2 inhibitors (e.g., ML385, molecule identified)^i^ Glutathione synthesis inhibition (e.g., Epalrestat, mouse model)^j^ mSWI/SNF inhibitors (cell lines, especially PC9‐OR cells)	[163, 203, 229]
	SMARCA4‐mutant	Inactivating mutation (e.g., Class I^k^)	PROTAC‐mediated degradation (e.g., A947, cell lines, and mouse model)^l^ HDAC inhibitors (preclinical studies)^m^ SMARCA2‐selective targeted protein degrader (e.g., PRT3789)^n^ CRISPR‐Cas9 (mouse models)^o^	[231, 232, 233, 234, 235, 236]
Targeting bypass pathway	MET	PI3K/AKT pathway activation	Combination (osimertinib plus savolitinib)^p^ Combination (amivantamab plus lazertinib)^q^ Bispecific antibody (e.g., bafisontamab/EMB‐01)^r^	[237, 238, 252]
	HER2		Covalent HER2 inhibitor (e.g., Zongertinib/BI 1810631)^s^	[239]
	HER3		Antibody‐drug conjugate (e.g., Patritumab deruxtecan/HER3‐DXd)^t^	[240]
	AXL	PLCγ‐PKC‐mTOR pathway activation	Type I inhibitors (e.g., ONO‐7475, CB469, pf‐02341066/Crizotinib)^u^ Type II inhibitors (e.g., BMS‐907351/Cabozantinib, RXDX‐106/CEP‐40783, MGCD516/Sitravatinib)^v^	[241, 242, 243, 244, 245, 246, 247]

^a^ Regimen adjustment based on co‐mutation profile: acquired PACC mutations coexisting with canonical primary EGFR mutations retain sensitivity to second‐generation TKIs while conferring allele‐specific resistance to third‐generation TKIs.

^b^ PACC: mutations on the interior surface of the ATP‐binding pocket or the C‐terminal end of the αC‐helix.

^c^ T790M‐like‐3S: T790M‐like mutants third‐generation TKI sensitive.

^d^ T790M‐like‐3R: complex mutations comprising T790M and a known drug‐resistance mutation.

^e^ Amivantamab: A retrospective real‐world study with an external control cohort comparing CHRYSALIS phase I trial (NCT02609776) versus real‐world therapies derived from three US‐based databases (ConcertAI, COTA, and Flatiron), and the most common therapies were nonplatinum chemotherapy, immunotherapy, EGFR‐TIKs, and platinum‐based chemotherapy.

^f^ Mobocertinib: A phase I/II single‐arm clinical trial, external controls came from real‐world data (RWD) included platinum‐pretreated patients.

^g^ EXCLAIM‑2: Mobocertinib's efficacy was not superior to platinum‐based chemotherapy for first‐line treatment of patients with *EGFR* ex20ins+ advanced/metastatic NSCLC.

^h^ Zipalertinib: a phase I/II open‐label trial (REZILIENT1, NCT04036682) enrolling patients with advanced/metastatic *EGFR* ex20ins‐mutant NSCLC treated with platinum‐based chemotherapy with/without amivantamab.

^i^ ML385: a probe molecule, in combination with carboplatin, showed antitumor activity in preclinical NRF2‐activated NSCLC models.

^j^ Epalrestat: Pharmacological inhibition (e.g., epalrestat) restored EGFR‐TKI sensitivity and delayed resistance in lung cancer patient‐derived xenograft mice.

^k^ Class I: SMARCA4 Class I mutations are truncating or loss‐of‐function variants (e.g., nonsense, frameshift, splice‐site, or large deletion).

^l^ A947: a selective SMARCA2 proteolysis‐targeting chimera molecule (PROTAC), translating to potent activity in SAMRCA4 mutant models.

^m^ HDAC inhibitors: Histone deacetylase inhibitors.

^n^ PRT3789: a clinical‐stage SMARCA2‐selective targeted protein degrader, inducing robust tumor growth inhibition and regression in SMARCA4‐deficient models.

^o^ CRISPR‐Cas9: a precise and highly efficient gene‐editing technology.

^p^ Osimertinib plus savolitinib: The phase 1b TATTON (NCT02143466) and phase 2 SAVANNAH (NCT03778229) studies laid the foundation for the SAFFRON study (NCT05261399), which is designed to evaluate the efficacy and safety of osimertinib plus savolitinib.

^q^ Amivantamab plus lazertinib: The CHRYSALIS trial (NCT02609776) is designed to analyze the potential for improved antitumor activity by combining amivantamab and Lazertinib in patients with *EGFR*‐mutant NSCLC.

^r^ EMB‐01: a bispecific antibody, designed to simultaneously target EGFR and receptor tyrosine kinase Met (c‐Met), shows potent antitumor activity across multiple tumor models.

^s^ BI 1810631: a covalent HER2 inhibitor, exhibits potent antitumor activity in HER2‐driven human NSCLC xenograft models.

^t^ HER3‐DXd: an antibody‐drug conjugate, demonstrated clinically meaningful and durable responses in a phase 2 study (NCT04619004), and its phase 3 trial (NCT05338970) is currently ongoing.

^u^ Type I inhibitors: AXL inhibitors bind to the active conformation of the AXL DFG motif.

^v^ Type II inhibitors: AXL inhibitors bind to the DFG residue in inactive AXL.

Furthermore, histological transformation, cancer stem cell subpopulations, the tumor immune microenvironment, epigenetic reprogramming, ferroptosis, and additional mechanisms collectively contribute to acquired resistance to EGFR‑TKIs in NSCLC [248, 249, 250, 251]. The SMARCA4‐EGFR crosstalk acts as a pivotal epigenetic driver of osimertinib resistance. Elucidating this regulatory interplay demands an integrated understanding of SMARCA4 loss‐of‐function mutations and the modulatory influences exerted on SMARCA4 by multiple signaling cascades. Exploring these dual regulatory dimensions is essential not only for deciphering the molecular basis of SMARCA4 functional deficiency in *EGFR*‐mutant NSCLC, but also for establishing a novel mechanistic framework that guides the development of targeted combination therapies to overcome osimertinib resistance.

In recent years, therapeutic strategies against osimertinib resistance have achieved remarkable progress. In the context of EGFR‐dependent resistance, the structure‐function‐based classification of EGFR mutations offers a new paradigm for guiding targeted drug selection: second‐generation EGFR‐TKIs exhibit superior efficacy against PACC subtype mutations (e.g., C797S, G719X), while ALK/PKC inhibitors are effective against T790M‐like‐3R compound mutations. For EGFR Ex20ins, a critical driver of both primary and acquired resistance, amivantamab, mobocertinib, and zipalertinib have demonstrated meaningful clinical benefit in platinum‐pretreated patients, addressing an important therapeutic gap for this subgroup. In SMARCA4‐related epigenetic resistance, exploiting synthetic lethal vulnerabilities has emerged as a promising direction. SMARCA2‐selective degraders and PROTAC molecules show potent antitumor activity in SMARCA4‐deficient models, while HDAC inhibitors and NRF2 inhibitors can restore osimertinib sensitivity by reversing chromatin remodeling defects and oxidative stress dysregulation. Additionally, chemo‑immunotherapy combinations markedly improve outcomes in SMARCA4‐deficient NSCLC patients, overcoming the partially immunosuppressive phenotype induced by SMARCA4 loss. Regarding bypass pathway activation, dual‐target inhibition and novel biologic therapeutics have become central research priorities: the combination of osimertinib and savolitinib effectively suppresses MET‐amplified resistance, the EGFR/c‐MET bispecific antibody (e.g., EMB‐01) achieves a high objective response rate across multiple tumor models [252], and HER3‐DXd exhibits broad antitumor activity across diverse EGFR‐TKI resistance mechanisms. AXL inhibitors are currently under preclinical and clinical evaluation, and their combination with EGFR‐TKIs is expected to reverse AXL‐mediated resistance.

Despite these advances, overcoming osimertinib resistance continues to face multiple critical challenges in both basic research and clinical translation. First, the spatiotemporal heterogeneity of resistance mechanisms remains a major clinical obstacle: intratumoral heterogeneity and clonal evolution give rise to the coexistence of multiple resistance mechanisms within a single patient, and current tissue biopsy and conventional genomic profiling techniques are unable to dynamically and comprehensively capture the full spectrum of resistance alterations. Second, the development of novel targeted agents encounters persistent bottlenecks: the majority of AXL inhibitors under investigation lack high specificity, leading to off‐target toxicities, and to date, no selective AXL‐targeting therapy has reached FDA approval [253]; fourth‐generation EGFR‐TKIs designed to target C797S‑containing compound mutations are still in the early‑stage research; and the first‐line efficacy of Ex20ins‐specific agents has not yet proven superior to platinum‐based chemotherapy, necessitating further optimization of drug design and combination strategies. Third, SMARCA4 deficiency in EGFR‐mutant NSCLC promotes the acquisition and maintenance of osimertinib resistance, and in already resistant cells, SMARCA4 functions as a critical sustainer of the resistant phenotype. However, the functional complexity of SMARCA4 mutations and the lack of predictive biomarkers limit clinical application: the biological functions of SMARCA4 class II missense mutations remain incompletely defined, and the correlation between mutation subtypes (homozygous truncation, heterozygous deletion) and therapeutic response to synthetic lethality drugs or immunotherapy awaits validation in large‐ scale cohort studies. Thus, exploiting SMARCA4 deficiency‐related vulnerabilities through TKI re‐sensitization or synthetic lethality emerges as a novel therapeutic paradigm for resistant EGFR‐mutant NSCLC and opens new therapeutic avenues. However, challenges persist: the functional complexity of nontruncating SMARCA4 mutations that retain partial activity, coupled with the preclinical status of SMARCA2/YAP1 inhibitors, underscores the need for predictive biomarkers. Such biomarkers would correlate SMARCA4 mutation type and expression levels with signaling profiles and clinical outcomes, and their incorporation demands further clinical validation to realize precision oncology.

In summary, osimertinib resistance in EGFR‐mutated NSCLC constitutes a multilayered, highly interrelated process that cannot be overcome by any single therapeutic strategy. Advancing precision oncology for this disease requires the close integration of basic research and clinical practice: from the in‐depth elucidation of the molecular mechanism of resistance and the development of high‐resolution diagnostic technologies, to the design of personalized combination regimens informed by individual resistance phenotypes, and the rapid translation of novel therapeutic strategies into clinical application. With continuous progress in omics technology, drug development, and clinical research, it is anticipated that real‐time monitoring, precise molecular classification, and individualized treatment of osimertinib resistance will become achievable, ultimately improving long‐term PFS and OS in patients with EGFR‐mutated NSCLC.

## Author Contributions

S.Q.F. designed the research. J.F.G. drafted the manuscript. S.Q.F. and Q.Y.W. revised the manuscript. All authors read and approved the final manuscript.

## Funding

The work, which included data collection, sample processing, and data analysis, was funded by the National Natural Sciences Foundation of China (No. 81972838; 82272722).

## Conflicts of Interest

The authors declare no conflicts of interest.

## Ethics Approval

The authors have nothing to report.

## Supporting information




**Table S1**: Clinical trials of recent advances in distinct types of EGFR‐TKI resistance.

## Data Availability

The authors have nothing to report.
