# Supplementary material for: Osimertinib Resistance in EGFR‐Mutated Non–Small Cell Lung Cancer: Mechanisms and Therapeutic Strategies
Source: MedComm (2020). 2026 Jul 2;7(7):e70823. doi: 10.1002/mco2.70823 (PMC13329268; doi:10.1002/mco2.70823)
Supplement: Supplementary file 1 — Table S1: Clinical trials of recent advances in distinct types of EGFR‐TKI resistance. [file MCO2-7-e70823-s001.docx]

**Osimertinib resistance in EGFR-mutated non-small cell lung cancer: mechanisms and therapeutic strategies**

**Junfeng Guo^1, 2^, Qiuyuan Wen^1, 2^, Songqing Fan^1, 2*^**

1. Department of Pathology, The Second Xiangya Hospital, Central South University, Changsha, Hunan, 410011, China

2. Hunan Clinical Medical Research Center for Cancer Pathogenic Genes Testing and Diagnosis, Changsha, Hunan, 410011, China

*Corresponding author: Songqing Fan, Department of Pathology, The Second Xiangya Hospital, Central South University, Changsha, Hunan, 410011, China. E-mail address: songqingfan@csu.edu.cn

**Table S1 Supplementary Information: Clinical Trials of Recent Advances in Distinct Types of EGFR-TKI Resistance**

| Type | Subtype/  Target | Investigational product | NCT No./Study types | Status | Phases | Preliminary findings | Reference |
| --- | --- | --- | --- | --- | --- | --- | --- |
| EGFR-Dependent | Ex20ins-FL | Amivantamab | NCT02609776 vs RWD | Completed | | Improved outcomes^a^ | 1 |
|  |  | Mobocertinib | NCT02716116 vs RWD | Completed | | Improved outcomes^b^ | 2 |
|  |  |  | NCT04129502 (EXCLAIM-2) | Terminated | III | No superior efficacy^c^ | 3 |
|  |  | Amivantamab vs Mobocertinib | MAIC^d^:  NCT02609776 vs NCT02716116 | Completed | | Similar efficacy^e^ | 4 |
|  |  | Zipalertinib | NCT04036682 (REZILIENT1) | Completed | I/II | Meaningful efficacy^f^ | 5 |
| Targeting Bypass Pathway | MET | Osimertinib plus Savolitinib | NCT05261399 (SAFFRON) | Ongoing | III | Exploratory analysis^g^ | 6 |
|  |  | Amivantamab plus lazertinib | NCT02609776 (CHRYSALIS) | Ongoing | I | Promising activity^h^ | 7 |
|  | HER3 | Patritumab deruxtecan/  HER3-DXd | NCT04619004 (HERTHENA-Lung01) | Completed | II | Meaningful efficacy^i^ | 8 |

a Improved outcomes of Amivantamab: Amivantamab achieved higher ORR (40% vs 16%), longer median PFS (8.3 vs 2.9months), longer median TTNT (14.8 vs 4.8months), and longer median OS (22.8 vs 12.8months) compared with the RWD group (ConcertAI, COTA, and Flatiron) in platinum-pretreated patients with advanced *EGFR* ex20ins-mutant NSCLC
b Improved outcomes of Mobocertinib: Mobocertinib achieved higher cORR (35.1% vs 11.9%), longer median PFS (7.3 vs 3.3 months), longer median OS (24.0 vs 12.4months) compared with the RWD group (Flatiron Health database) in platinum-pretreated patients with advanced *EGFR* ex20ins-mutant NSCLC
c No superior efficacy of Mobocertinib: Based on interim analysis (cutoff: April 4, 2023), mobocertinib was not superior to platinum-based chemotherapy for first-line treatment of patients with *EGFR* ex20ins-mutant advanced/metastatic NSCLC, with similar median PFS [9.6 vs 9.6 months, 95% confidence interval (CI), 0.77-1.39]), comparable cORR (32% vs 30%), and non-inferior median OS (not estimable vs 30.0 months).
d MAIC: Matching-adjusted indirect comparison
e Similar efficacy of Amivantamab vs Mobocertinib: The weighted OR (95% CI) for independent review committee (IRC)-assessed and investigator (INV)-assessed confirmed ORR (cORR) were 0.56 (0.30-1.04) and 0.98 (0.53-1.82) respectively, and IRC-assessed PFS was 0.74 (0.51-1.07), OS was 0.92 (0.57-1.48), and INV-assessed duration of response (DoR) was 0.59 (0.30-1.18)
f Meaningful efficacy of Zipalertinib: At data cutoff (December 10, 2024), the cORR and median DOR (95% CI) of patients received zipalertinib was 35.2% (28.2-42.8) and 8.8months (8.3-12.7) respectively, and the former exceeding the historical benchmark of 20% with chemotherapy
g Exploratory analysis of NCT05261399 (SAFFRON): The NCT05261399 (SAFFRON) trial is undergoing, which is designed to assess the efficacy and safety of the savolitinib-osimertinib combination with platinum-based chemotherapy in *EGFR*-mutated NSCLC patients with *MET* overexpression/amplification who have failed osimertinib treatment
h Promising activity of Amivantamab plus Lazertinib: Amivantamab plus lazertinib had a safety profile consistent with monotherapy, achieving an ORR of 36% (95% CI 22-51), median DOR of 9.6 months and median PFS of 4.9 months. Additionally, NCT04487080 (MARIPOSA), NCT04988295 (MARIPOSA-2), NCT05388669 (PALOMA-3) are ongoing
i Meaningful efficacy of Patritumab deruxtecan: The safety profile of Patritumab deruxtecan was manageable and tolerable. As of May 18, 2023, cORR was 29.8 (23.9-36.2), median study duration was 18.9months (range, 14.9-27.5), median PFS was 5.5months, and median OS was 11.9months

**Declarations
Acknowledgements**

Not applicable.
**Authors' contributions**

SQF designed the research. JFG drafted the manuscript. SQF and QYW revised the manuscript. All authors read and approved the final manuscript.
**Conflicts of interest**

The authors declare no conflict of interest.

**Data availability statement**

Not applicable.

**Ethics approval**

Not applicable.

**Funding information**

The work included data collection, sample processing, and data analysis, was funded by the National Natural Sciences Foundations of China (No. 81972838; 82272722).

**References**

1. Minchom A, Viteri S, Bazhenova L, et al. Amivantamab compared with real-world therapies in patients with advanced non-small cell lung cancer harboring EGFR exon 20 insertion mutations who progressed after platinum-based chemotherapy. *Lung Cancer*. 2022; 168:74-82.
2. Ou SI, Lin HM, Hong JL, et al. Comparative effectiveness of mobocertinib and standard of care in patients with NSCLC with EGFR exon 20 insertion mutations: An indirect comparison. *Lung Cancer*. 2023; 179:107186.
3. Jänne PA, Wang BC, Cho BC, et al. First-Line Mobocertinib Versus Platinum-Based Chemotherapy in Patients With EGFR Exon 20 Insertion-Positive Metastatic Non-Small Cell Lung Cancer in the Phase III EXCLAIM-2 Trial. *J Clin Oncol*. 2025; 43(13):1553-1563.
4. Ou SI, Prawitz T, Lin HM, et al. Efficacy of Mobocertinib and Amivantamab in Patients With Advanced Non-Small Cell Lung Cancer With EGFR Exon 20 Insertions Previously Treated With Platinum-Based Chemotherapy: An Indirect Treatment Comparison. *Clin Lung Cancer*. 2024; 25(3):e145-e152.e3.
5. Piotrowska Z, Passaro A, Nguyen D, et al. Zipalertinib in Patients With Epidermal Growth Factor Receptor Exon 20 Insertion-Positive Non-Small Cell Lung Cancer Previously Treated With Platinum-Based Chemotherapy With or Without Amivantamab. *J Clin Oncol*. 2025; 43(21):2387-2397.
6. Lu S, Xu W, Telaranta-Keerie A, et al. EP08.02-138 SAFFRON: Ph3 Savolitinib + Osimertinib vs Chemotherapy in EGFRm NSCLC with MET Overexpression/Amplification Post-Osimertinib. *J Thorac Oncol*. 2022, 17, S468–S469.
7. Cho BC, Kim DW, Spira AI, et al. Amivantamab plus lazertinib in osimertinib-relapsed EGFR-mutant advanced non-small cell lung cancer: a phase 1 trial. *Nat Med*. 2023; 29(10):2577-2585.
8. Yu HA, Goto Y, Hayashi H, et al. HERTHENA-Lung01, a Phase II Trial of Patritumab Deruxtecan (HER3-DXd) in Epidermal Growth Factor Receptor-Mutated Non-Small-Cell Lung Cancer After Epidermal Growth Factor Receptor Tyrosine Kinase Inhibitor Therapy and Platinum-Based Chemotherapy. *J Clin Oncol*. 2023; 41(35):5363-5375.
